# White spot syndrome virus IE1 protein hijacks the host pentose phosphate pathway to fuel viral replication

**DOI:** 10.1371/journal.ppat.1013913

**Published:** 2026-01-27

**Authors:** Jia Zhang, Kaiyu Lu, Jinghua Zhu, Jude Juventus Aweya, Yueling Zhang, Defu Yao

**Affiliations:** 1 Institute of Marine Sciences and Guangdong Provincial Key Laboratory of Marine Biology, Shantou University, Shantou, China; 2 Department of Food and Human Nutritional Sciences, University of Manitoba, Winnipeg, Manitoba, Canada; Pirbright Institute, UNITED KINGDOM OF GREAT BRITAIN AND NORTHERN IRELAND

## Abstract

Viruses frequently reprogram host metabolism to support their replication. The pentose phosphate pathway (PPP), a key regulator of nucleotide synthesis and redox balance, is often targeted during infection. While PPP activation is well-known proviral mechanism in vertebrates, how this process occurs in invertebrate hosts remains unclear. Here, using the white spot syndrome virus (WSSV) and its penaeid shrimp host as a model, we identify a previously unrecognized viral strategy that directly reprograms the PPP through host-viral protein interaction. WSSV infection strongly induced the expression of key PPP enzymes, including glucose-6-phosphate dehydrogenase (G6PD) and transketolase-like 2 (TKTL2), and enhanced TKTL2 enzymatic activity. This activation increased the production of nicotinamide adenine dinucleotide phosphate (NADPH) and ribose-5-phosphate (R5P), two critical PPP metabolites. Functional assays confirmed that the PPP is essential for efficient WSSV replication, as knockdown or pharmacological inhibition of G6PD or TKTL2 significantly attenuated viral load and improved host survival. Mechanistically, the viral immediate-early protein IE1 was found to directly bind to the C-terminal region of TKTL2 (TKTL2-C1, residues 500–555), and enhance its enzymatic activity. This interaction promoted PPP flux, boosted NADPH and R5P biosynthesis, and suppressed reactive oxygen species (ROS) accumulation. Supplementation with NADPH, R5P, or a ROS scavenger restored viral replication defects caused by IE1 knockdown. Moreover, the IE1-binding fragment TKTL2-C1 acted as a competitive inhibitor that disrupted the IE1-TKTL2 interaction, decreased PPP flux, and reduced viral replication. Together, these findings demonstrate that WSSV IE1 directly activates host TKTL2 to rewire pentose phosphate metabolism, revealing a novel metabolic mechanism of viral pathogenesis and identifying the PPP as a potential target for antiviral intervention in aquaculture.

## Author summary

Viruses act like cellular hijackers, taking control of their host’s machinery to make more copies of themselves. One important part of this takeover involves reprogramming the host’s metabolism, especially a process called the pentose phosphate pathway (PPP). This pathway provides the molecular building blocks and reducing power needed for viral replication and defense against stress. Although many human viruses are known to exploit the PPP, much less is known about how invertebrate viruses, such as those infecting shrimp, achieve this. In this study, we examined how white spot syndrome virus (WSSV), a major pathogen in shrimp aquaculture, manipulates the PPP. We discovered that the viral immediate-early protein IE1 binds directly to the shrimp enzyme TKTL2, increasing its enzymatic activity. This interaction enhances PPP flux, elevates NADPH and R5P levels, and lowers reactive oxygen species (ROS), creating a cellular environment that favors viral replication. Our findings uncover a direct and efficient mechanism of metabolic hijacking, where a viral protein activates a key host enzyme to meet its biosynthetic and redox demands. This work highlights the PPP as a promising metabolic target for developing antiviral strategies against WSSV.

## Introduction

Viruses are obligate intracellular parasites that rely entirely on host cellular machinery and resources for replication. This dependence requires a fundamental remodeling of host metabolism, which is a central aspect of virus–host interactions. During long-term co-evolution, hosts have developed defense mechanisms that limit viral access to essential metabolites or induce the synthesis of antiviral compounds to restrict viral propagation [1,2]. In response, viruses have evolved sophisticated counterstrategies to hijack metabolic pathways, redirecting key carbon sources such as glucose and glutamine toward their own biosynthetic and energy needs [3–5]. Understanding these virus-induced metabolic alterations provides important insights into viral pathogenesis and offers potential metabolic targets for antiviral intervention.

The pentose phosphate pathway (PPP) is a central metabolic branch connecting carbohydrate metabolism with nucleotide biosynthesis, functioning in close coordination with glycolysis and the tricarboxylic acid (TCA) cycle [6]. Diverging from glycolysis at glucose-6-phosphate (G6P), the PPP consists of two functionally distinct phases. The oxidative phase, initiated and rate-limited by glucose-6-phosphate dehydrogenase (G6PD), generates nicotinamide adenine dinucleotide phosphate (NADPH), which supplies reducing power for biosynthetic reactions and antioxidant defense. The subsequent non-oxidative phase, catalyzed mainly by transketolase (TKT) and transketolase-like protein (TKTL1 and TKTL2), enables the reversible conversion of sugar phosphates to produce ribose-5-phosphate (R5P) for nucleotide synthesis, while also generating fructose-6-phosphate (F6P) and glyceraldehyde-3-phosphate (GAP) that can re-enter glycolysis [7,8]. Given its central roles in biosynthesis and redox homeostasis, many viruses have evolved to exploit the PPP. For example, Hepatitis B virus enhances G6PD expression through HBx-mediated activation of the transcription factor Nrf2 [9]. Similarly, Human Papillomavirus (HPV) activates the PPP by promoting G6PD dimer formation via inhibition of its lactylation, thereby providing anabolic support for cervical cancer cell proliferation [10]. In contrast, SARS-CoV-2 depends on the non-oxidative branch, where elevated transketolase (TKT) activity is essential for viral replication, and TKT inhibition strongly suppresses infection [11]. Despite these advances, most current knowledge of virus-induced PPP regulation comes vertebrate systems, while little is known about how viruses infecting invertebrate hosts manipulate this pathway.

White spot syndrome virus (WSSV), the only member of the genus *Whispovirus*, is a major pathogen that causes significant economic losses in global shrimp aquaculture [12]. This large DNA virus extensively reprograms host metabolism to support its replication [13]. WSSV induces a Warburg-like metabolic shift during the viral genome replication stage (12 h post-infection, hpi), increasing glycolytic flux through the activation of PI3K-Akt-mTOR and CaMKK-AMPK-mTORC2 signaling pathways [14–17]. In addition to glycolysis, WSSV rewires glutamine metabolism by upregulating glutamate dehydrogenase (GDH) and aspartate aminotransferase (ASAT), thereby replenishing the TCA cycle and promoting *de novo* nucleotide synthesis. Both glucose and glutamine serve as key precursors for viral genome replication [18–20]. The virus also shows temporal regulation of lipid metabolism: it stimulates lipolysis and β-oxidation at 12 hpi to generate energy, followed by a PI3K–Akt–mTOR–HIF1α-mediated lipogenesis by 24 hpi, during which fatty acid synthase (FAS) upregulation produces long-chain fatty acids required for virion assembly [21,22]. In parallel, WSSV modulates the PPP to maintain redox homeostasis. The viral protein wsv220 binds to the Nrf2 repressor Keap1, disrupting the Nrf2-Keap1 complex and promoting Nrf2 nuclear translocation. This activates antioxidant genes such as G6PD, enhances PPP flux, increases NADPH production, and lowers reactive oxygen species (ROS) levels, thereby facilitating viral replication [23]. Complementing this transcriptional mechanism, our previous proteomic analysis revealed a physical interaction between the WSSV immediate-early protein IE1 and host transketolase-like 2 (TKTL2) [24], suggesting that WSSV may also directly activate the PPP to rapidly secure biosynthetic resources for its replication.

In this study, we demonstrate that WSSV infection activates the PPP in the shrimp *Penaeus vannamei* and identify the viral immediate-early protein IE1 as a critical regulator of this process. We show that IE1 directly binds to the C-terminal region of TKTL2 and enhances its enzymatic activity, thereby stimulating PPP flux. This IE1–TKTL2 interaction increases NADPH and R5P production while reducing ROS accumulation, establishing a metabolic environment favorable for viral replication. Our findings reveal a novel mechanism by which WSSV, through its IE1 protein, hijacks the host PPP to promote viral propagation, highlighting this pathway as a promising target for antiviral intervention.

## Results

### The pentose phosphate pathway (PPP) is activated upon WSSV infection

**Fig 1:**
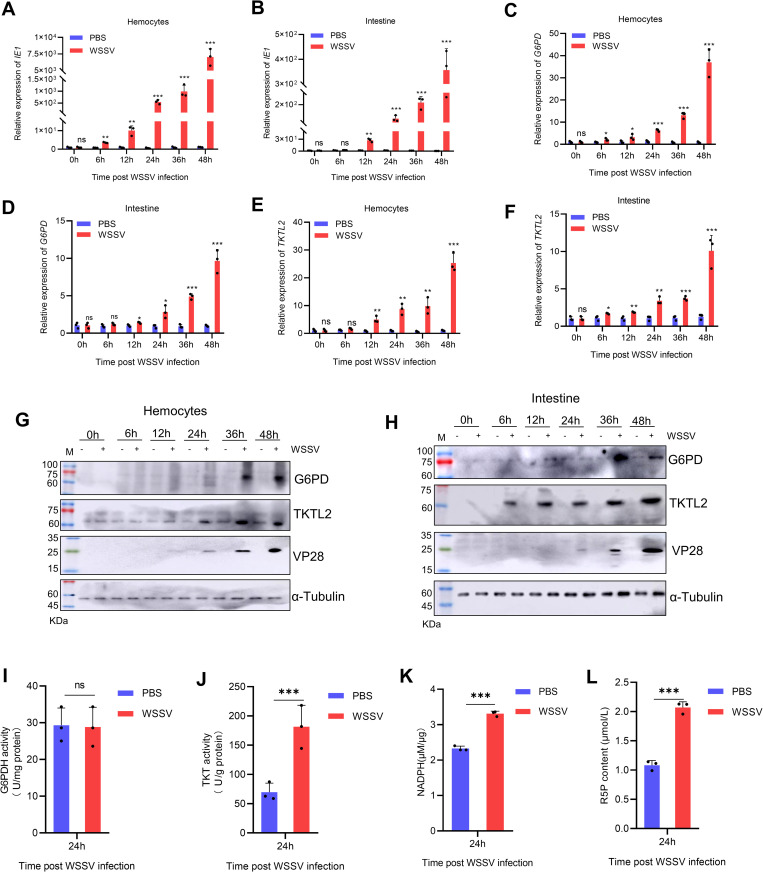
WSSV infection activates the pentose phosphate pathway (PPP) in shrimp. (A–F) Temporal expression profiles of viral and PPP-related genes. Hemocytes and intestinal tissues were collected from shrimp at 0, 6, 12, 24, 36, and 48 h post-injection (hpi) with WSSV or PBS. The mRNA levels of the viral gene IE1 (A, B) and the host genes G6PD (C, D) and TKTL2 (E, F) were determined by qPCR. (G, H) Western blot detection of VP28, TKTL2, and G6PD proteins in hemocytes (G) and intestinal tissues (H) at the indicated time points. (I, J) Enzymatic activity of TKT and G6PD in hemocytes collected at 24 hpi. WSSV- or PBS-injected lysates were used to measure the activity of G6PD (I) and TKT (J). (K, L) Quantification of PPP metabolites. Hemocytes collected at 24 hpi were analyzed for (K) NADPH levels using a detection kit and (L) R5P levels by LC-MS. Data are expressed as mean ± SD from three biological replicates. Asterisks indicate statistically significant differences compared with PBS control. *p< 0.05, **p < 0.01, and ***p < 0.001. ns, not significant.

To examine whether the PPP is involved in WSSV infection, we first analyzed its temporal activation profile. The expression and enzymatic activity of two key PPP enzymes, G6PD and TKTL2, were measured, together with the pathway metabolites NADPH and R5P, at different times post-infection. Successful viral infection was confirmed by the progressive increase in expression of the immediate-early gene IE1 and the late structural gene VP28 in both hemocytes and intestinal tissue (Fig 1A and 1B). Compared with PBS-injected controls, WSSV-infected shrimp showed a significant upregulation of G6PD and TKTL2 at both the mRNA and protein levels (Fig 1C-1H). In hemocytes, TKTL2 enzymatic activity increased significantly at 24 h post-infection (hpi), whereas G6PD activity showed no notable change (Fig 1I and 1J). Consistent with this, intracellular levels of NADPH and R5P were markedly elevated at 24 hpi (Fig 1K and 1L). Together these findings indicate that WSSV infection activates the PPP in shrimp, primarily through the non-oxidative branch mediated by TKTL2.

### The PPP is required for efficient WSSV infection

**Fig 2:**
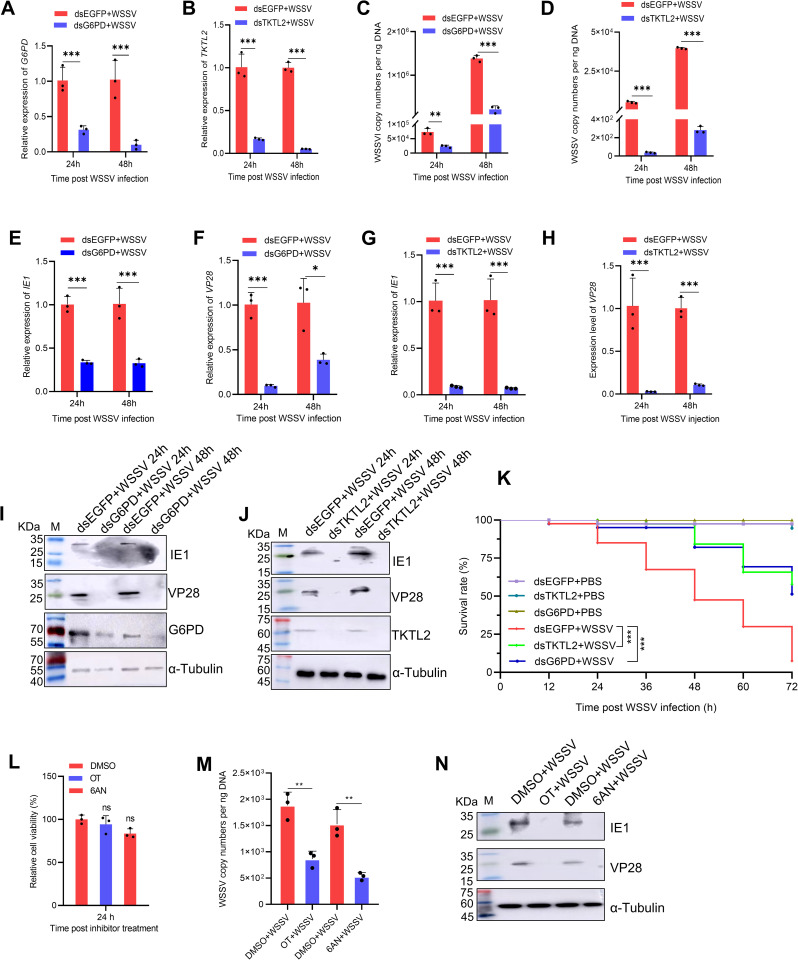
The PPP is essential for WSSV infection. (A–J) Effects of G6PD and TKTL2 knockdown on WSSV infection. Shrimp were injected with dsRNA targeting G6PD (dsG6PD), TKTL2 (dsTKTL2), or control dsEGFP, followed by WSSV challenge at 48 h post-injection. Hemocytes collected at 24 and 48 hpi were analyzed for (A, B) knockdown efficiency, (C, D) viral load, (E–H) mRNA expression of viral genes (IE1 and VP28), and (I, J) viral protein levels. (K) Survival analysis of shrimp after gene knockdown and WSSV or PBS challenge. (L–N) Effects of PPP enzyme inhibitors on WSSV infection. Shrimp were injected with 2 μM oxythiamine (OT; TKTL2 inhibitor), 20 μM 6-aminonicotinamide (6-AN; G6PD inhibitor), or DMSO control. Cytotoxicity was evaluated by CCK‑8 assay (L). At 2 h post-injection, shrimp were challenged with WSSV, and hemocytes were collected at 24 hpi for analysis of (M) viral load, and (N) viral protein expression. Data are shown as mean ± SD from three biological replicates. *p < 0.05, **p < 0.01, ***p < 0.001; ns, not significant.

To determine whether the PPP plays a functional role in WSSV replication, we performed RNA interference (RNAi) to silence key PPP enzymes. Knockdown of either G6PD or TKTL2 led to a significant reduction in viral loads compared with the dsRNA control group after WSSV challenge (Fig 2A-2D). Correspondingly, the transcript and protein levels of the viral genes IE1 and VP28 were markedly decreased following G6PD or TKTL2 silencing (Fig 2E-2J). Moreover, shrimp with suppressed PPP gene expression exhibited significantly higher survival rates during infection (Fig 2K). To further validate observations pharmacologically, we applied two metabolic inhibitors: 6-aminonicotinamide (6AN), a G6PD inhibitor, and oxythiamine (OT), a TKT inhibitor. Both compounds displayed no apparent cytotoxicity but caused significant reduction in viral load and in the expression of IE1 and VP28 (Fig 2L-2N). Collectively, these results demonstrate that activation of the PPP, particularly via G6PD and TKTL2, is essential for efficient WSSV replication and host infection.

### IE1 directly binds to the C-terminus of host TKTL2

**Fig 3:**
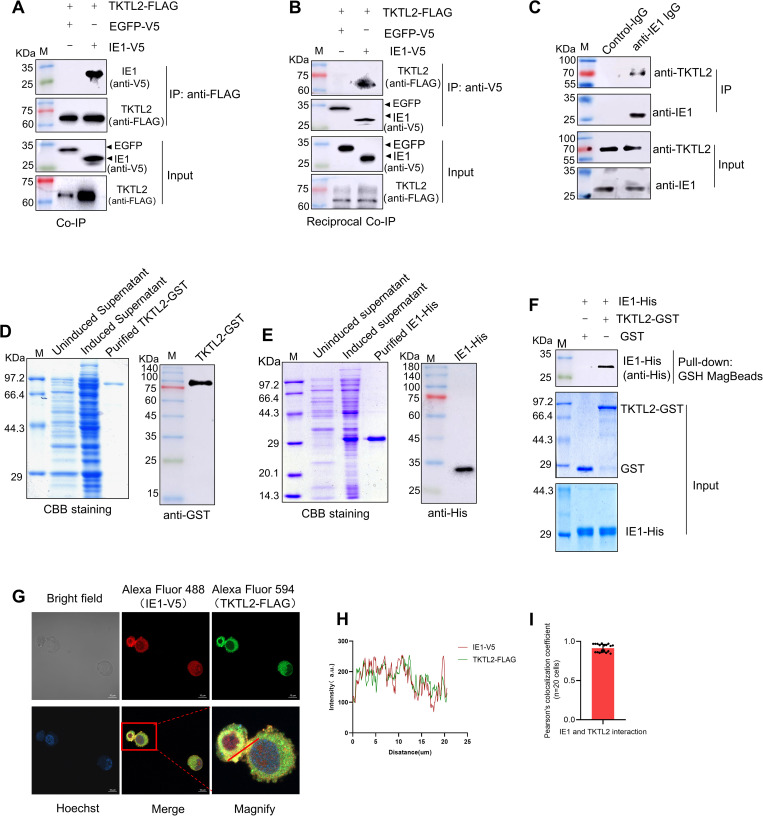
IE1 directly interacts with TKTL2. (A, B) Co-immunoprecipitation (Co‑IP) of IE1 and TKTL2 in transfected High Five cells. Cells co‑transfected with pIZ-FLAG-TKTL2 and either pIZ-V5-IE1 or pIZ-V5-EGFP were harvested at 48 h post‑transfection. Lysates were immunoprecipitated with anti‑FLAG magnetic beads (A) or anti‑V5 antibody coupled to protein A/G beads (B), followed by Western blot detection. (C) Endogenous Co‑IP in WSSV‑infected shrimp hemocytes collected at 24 hpi. Lysates were immunoprecipitated with anti‑IE1 antibody or control IgG, and analyzed with anti‑TKTL2 and anti‑IE1 antibodies. (D–F) GST pull‑down assay using recombinant proteins. (D, E) Expression and purification of TKTL2-GST and IE1-His in *E. coli*. (F) GST or TKTL2-GST bound to glutathione beads was incubated with His‑IE1, and bound proteins were detected by Western blot. (G-I) Subcellular co‑localization of TKTL2 and IE1 in High Five cells was visualized by confocal microscopy (G; scale bar, 10 μm). Co‑localization was quantified in ImageJ using fluorescence intensity profile (H) and Pearson’s correlation coefficient (I) analyses (n = 20 cells).

Previous proteomic analysis suggested a potential interaction between WSSV IE1 and the host enzyme TKTL2. We therefore hypothesized that WSSV may modulate the PPP through a direct interaction between these two proteins. To verify this, we performed Co-immunoprecipitation (co-IP) assays in High Five cells. V5-tagged IE1 (V5-IE1), but not the V5-tagged EGFP control (V5-EGFP), co-precipitated with FLAG-tagged TKTL2 (FLAG-TKTL2) (Fig 3A). Reciprocally, FLAG-TKTL2 was efficiently pulled down in immunoprecipitates containing V5-IE1 (Fig 3B).

To test whether this interaction occurs under physiological conditions, we performed co-IP in shrimp using custom-made antibodies. Endogenous TKTL2 was specifically detected in IE1 immunoprecipitates, but not in control IgG samples (Fig 3C). Next, to determine if the interaction is direct, purified GST-tagged TKTL2 (TKTL2-GST) and His-tagged IE1 (IE1-His) were subjected to an in vitro GST pull-down assay. IE1-His was captured by TKTL2-GST immobilized on glutathione magnetic beads, but not by GST alone (Fig 3F), confirming a direct binding between IE1 and TKTL2. Immunofluorescence imaging further showed a co-localization of IE1 and TKTL2 in the cytoplasm of High Five cells (Fig 3G-3I).

**Fig 4:**
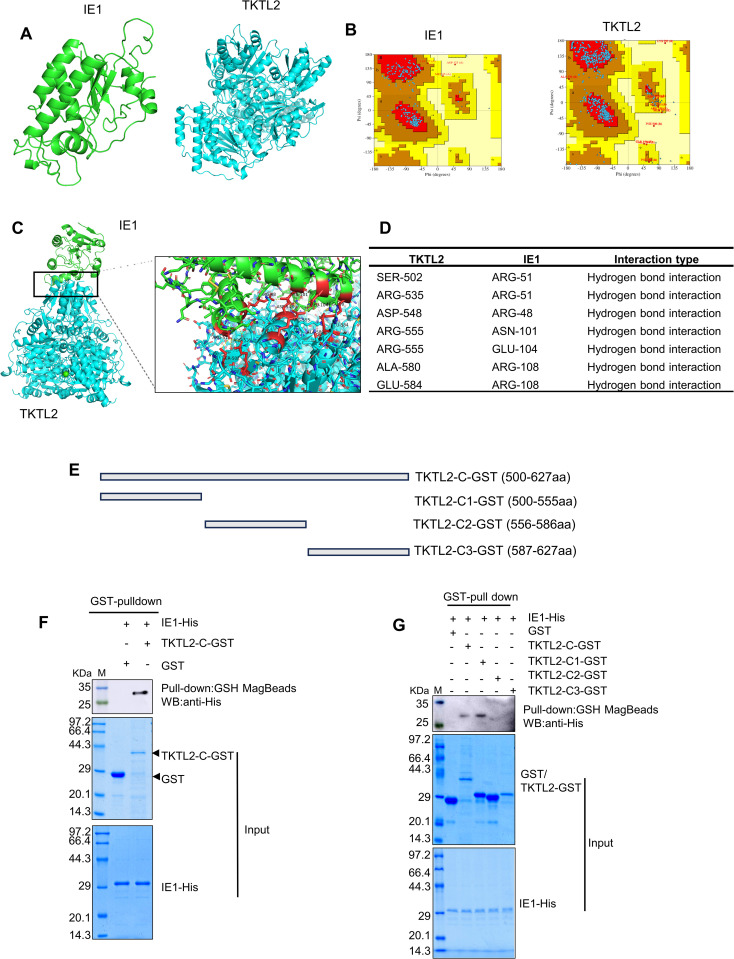
IE1 binds to the C-terminal region of TKTL2. (A, B) Structural modeling of IE1 and TKTL2 generated by SWISS-MODEL (A) and validated by Ramachandran plot (B). (C, D) Molecular docking of IE1 and TKTL2 performed with HDOCK (C), and predicted binding sites are summarized (D). (E) Schematic of constructed plasmids expressing the C-terminal region of TKTL2 (TKTL2-C-GST) and its subsegments (TKTL2-C1/C2/C3-GST) fused to GST. (F, G) GST pull-down validation of the IE1–TKTL2 interaction. Each GST-tagged protein was expressed, immobilized on glutathione magnetic beads, and incubated with purified IE1-His. Bound proteins were eluted and detected by anti-His Western blot.

To identify the interaction region, molecular docking analysis was performed. Homology models of IE1 and TKTL2 were generated, and Ramachandran plot analysis indicated that more than 99% of residues were within allowed regions, supporting model reliability (Fig 4A and 4B). Docking predictions using HDOCK2 indicated that IE1 interacts mainly with the C-terminal region of TKTL2, involving residues Ser502, Arg535, Asp548, Arg555, Ala580, and Glu584 (Fig 4C and 4D). To validate this prediction experimentally, the C-terminal fragment of TKTL2 (amino acids 500–627, TKTL2-C) was expressed as a GST‑fusion protein and tested for interaction with IE1 using GST pull‑down assay. The results confirmed that IE1 specifically binds to the C‑terminus of TKTL2 (Fig 4E and 4F). To map the binding site more precisely, this region was subdivided into three segments: TKTL2‑C1 (aa 500–555), TKTL2‑C2 (aa 556–586), and TKTL2‑C3 (aa 587–627). Among these, only TKTL2‑C1 retained the ability to bind IE1, defining it as the minimal IE1‑interaction region within TKTL2 (Fig 4G).

### IE1 activates TKTL2 enzymatic activity and promotes PPP flux

**Fig 5:**
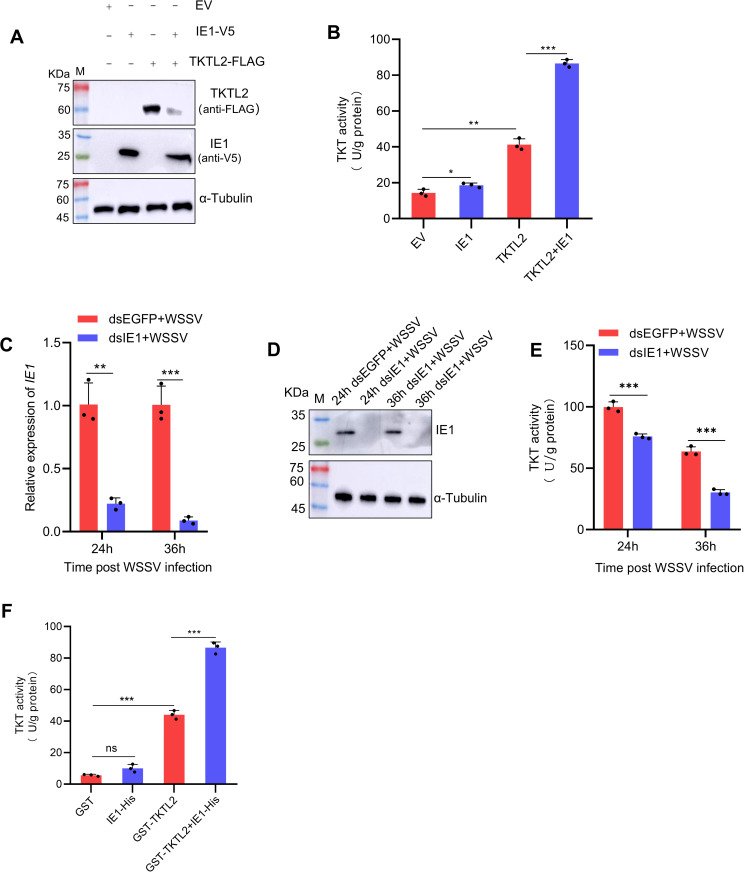
IE1 enhances the enzymatic activity of TKTL2. (A, B) Effect of IE1 overexpression on TKT activity in High Five cells transfected with empty vector pIZ‑V5‑His (EV), pIZ‑V5‑IE1, pIZ‑FLAG‑TKTL2, or both pIZ‑V5‑IE1 and pIZ‑FLAG‑TKTL2 constructs. (A) Western blot detection of protein expression; (B) TKT enzymatic activity measured using a commercial kit. (C–E) Effect of IE1 knockdown on TKT activity in shrimp. Shrimp injected with dsIE1 or dsEGFP were challenged with WSSV and hemocytes were collected at 24 and 36 hpi to assess (C, D) IE1 knockdown efficiency and (E) TKT enzymatic activity. (F) *In vitro* TKT assay showing direct enhancement of TKTL2 activity by IE1 using purified recombinant proteins. Data represent mean ± SD from three biological replicates. *p< 0.05, **p < 0.01, ***p < 0.001. ns, not significant.

We next examined whether the interaction between IE1 and TKTL2 affects the enzymatic activity of TKTL2. Overexpression of IE1 in High Five cells caused a slight increase in endogenous TKT activity compared with the control. When IE1 was co-expressed with shrimp TKTL2, TKT activity was significantly higher than in cells expressing TKTL2 alone (Fig 5A and 5B). To evaluate the physiological relevance of this effect, we analyzed TKT activity in shrimp after IE1 knockdown. Silencing of IE1 in WSSV-challenged shrimp led to significant reduction in TKT activity in hemocytes compared with the control group (Fig 5C-5E). Furthermore, in vitro enzymatic assays using recombinant proteins showed that recombinant TKTL2 alone displayed measurable TKT activity, and this activity was markedly enhanced when incubated with recombinant IE1 (Fig 5F). These results indicate that IE1 directly stimulates the enzymatic activity of TKTL2.

**Fig 6:**
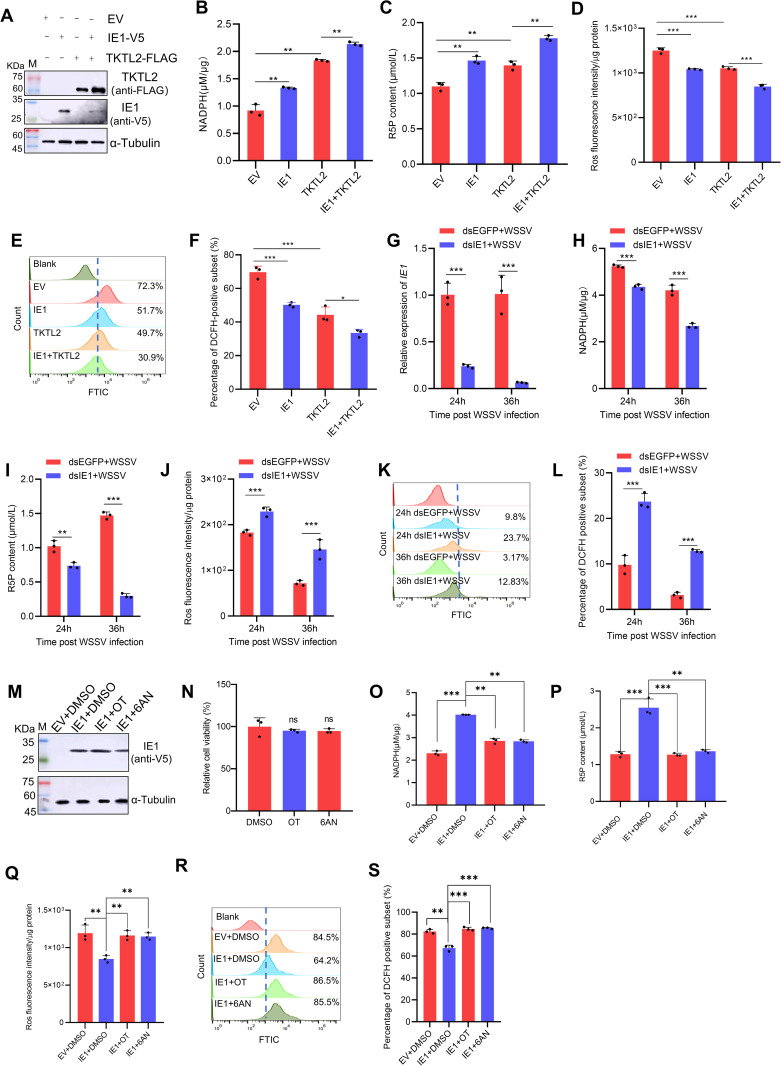
IE1 enhances PPP flux through interaction with TKTL2. (A-F) Effects of IE1 overexpression on PPP metabolites in High Five cells transfected with empty vector pIZ-V5‑His (EV), pIZ-V5-IE1, pIZ-FLAG-TKTL2, or both pIZ-V5-IE1 and pIZ-FLAG-TKTL2. (A) Western blot detection; (B) NADPH levels and (C) R5P levels; (D-F) ROS levels analyzed by microplate reader (D) and flow cytometry (E, F). (G-L) Effects of IE1 knockdown (G) in shrimp on (H) NADPH, (I) R5P, and (J-L) ROS levels. (M-S) Effects of IE1 overexpression on PPP flux under TKT and G6PD inhibition. High Five cells transfected with EV or pIZ-V5-IE1 were treated with 25 μM OT (TKTL2 inhibitor), 25 µM 6AN (G6PD inhibitor), or DMSO control. (M) Western blot detection; (N) Inhibitor cytotoxicity; (O) NADPH, (P) R5P, and (Q-S) ROS measurements. Data are presented as mean ± SD from three biological replicates. *p < 0.05, **p < 0.01, ***p < 0.001. ns, not significant.

To determine whether IE1 promotes the PPP through TKTL2 activation, we measured PPP-related metabolites and cellular redox status under IE1 overexpression or knockdown conditions. Co-expression of IE1 and TKTL2 in High Five cells significantly increased NADPH and R5P levels and decreased ROS compared with untransfected or TKTL2-only controls (Fig 6A-6F). In contrast, IE1 knockdown in WSSV-infected shrimp significantly reduced NADPH and R5P levels while increasing ROS accumulation compared with dsEGFP-treated controls (Fig 6G-6L). Moreover, treating IE1-overexpressing cells with the PPP inhibitors (6AN or OT) substantially decreased NADPH and R5P levels and restored ROS accumulation (Fig 6M-6S).

Together, these results demonstrate that IE1 enhances PPP flux by activating TKTL2, thereby promoting NADPH production and maintaining redox balance during WSSV infection.

### IE1 promotes WSSV infection through PPP metabolites

**Fig 7:**
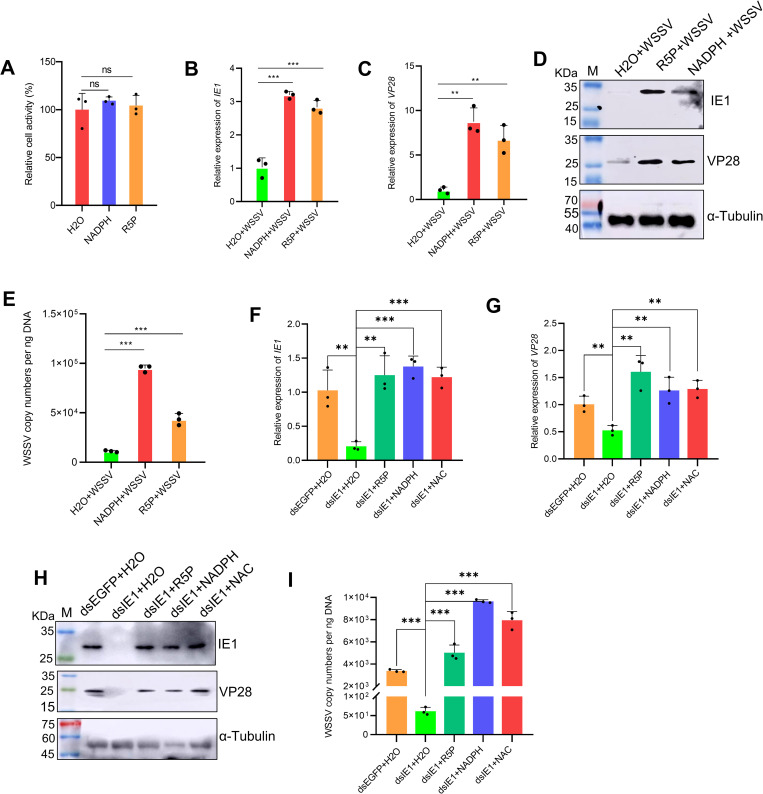
IE1 promotes WSSV infection through PPP metabolites. (A–E) Effect of R5P and NADPH supplementation on WSSV infection. Shrimp were injected with 10 μM R5P, NADPH, or DEPC‑H₂O as control. (A) Cytotoxicity at 10 μM evaluated by CCK‑8. At 2 h post‑injection, shrimp were challenged with WSSV and hemocytes were collected at 24 hpi to analyze (B, C) viral gene expression (IE1, VP28), (D) viral protein levels, and (E) viral load. (F–I) Rescue of WSSV replication following IE1 knockdown. Shrimp pretreated with dsIE1 or dsEGFP were co-injected with WSSV and one of the following: 10 μM NADPH, 10 μM R5P, 10 μM NAC, or DEPC-H₂O control. Hemocytes collected at 24 hpi were analyzed for (F, G) viral gene expression, (H) viral protein levels, and (I) viral load. Data are expressed as mean ± SD from three biological replicates. **p< 0.01, ***p< 0.001. ns, not significant.

Given that NADPH and R5P play essential roles in nucleotide biosynthesis and redox regulation, we hypothesized that these PPP-derived metabolites act as key mediators of the proviral effect of IE1. To test this idea, we first performed *in vivo* metabolite supplementation experiments. Administration of NADPH or R5P to WSSV-infected shrimp significantly increased the transcript and protein levels of the viral genes IE1 and VP28, as well as total viral loads, without causing detectable cytotoxicity (Fig 7A-7E).

To ascertain that IE1 promotes viral infection mainly by elevating these metabolites, we conducted a rescue experiments combining IE1 knockdown with supplementation of NADPH, R5P, or the ROS scavenger N-acetylcysteine (NAC). Supplementation with any of these compounds effectively restored viral replication that was impaired by IE1 silencing (Fig 7F-7I). Collectively, these data demonstrate that IE1 facilitates WSSV infection by augmenting the production of key PPP metabolites, thereby creating a favorable metabolic environment for viral propagation.

### TKTL2-C1 fragment competitively suppresses PPP activation and WSSV replication

**Fig 8:**
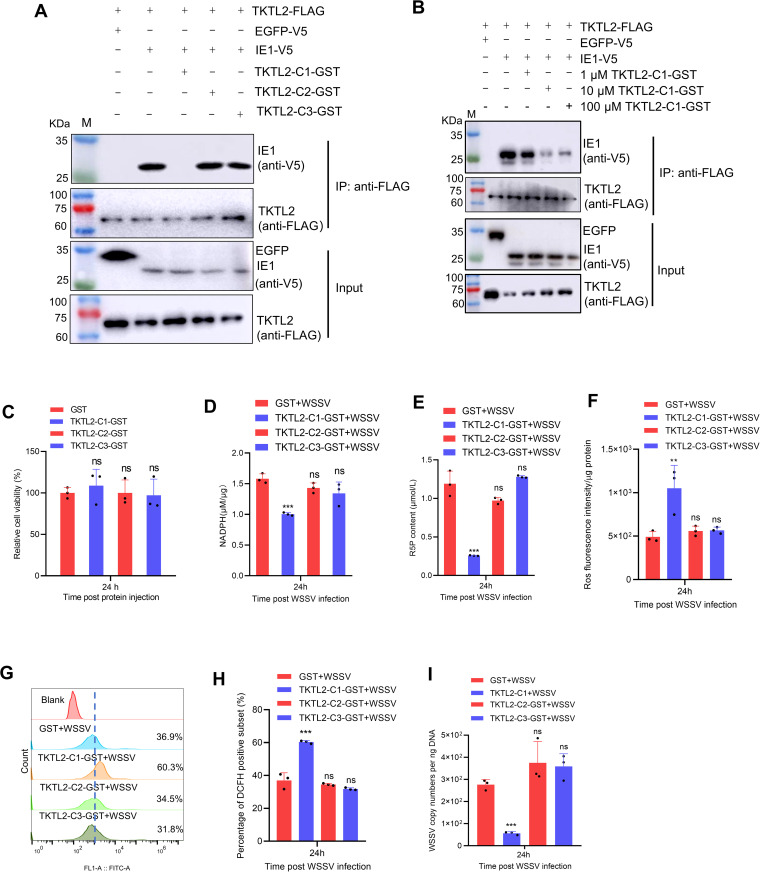
TKTL2‑C1 fragment competitively inhibits PPP activation and WSSV replication. (A–B) Effect of TKTL2‑C1 on the IE1–TKTL2 interaction. Lysates from cells co‑transfected with pIZ‑FLAG‑TKTL2 and pIZ‑V5‑IE1 were mixed with 100 μM of the indicated recombinant GST‑fusion proteins (TKTL2‑C1/C2/C3‑GST) (A) or with increasing concentrations of TKTL2‑C1‑GST (0, 1, 10 and 100 μM) (B), followed by co‑IP analysis. (C) Cytotoxicity of recombinant GST‑fusion proteins. Shrimp were injected with 10 μM GST or TKTL2‑C1/2/3‑GST; hemocytes were collected 24 h post‑injection for cell‑viability assay. (D–I) Impact of TKTL2‑C1 on PPP flux and viral infection. Shrimp were injected with 10 μM of the indicated GST‑fusion proteins together with WSSV; hemocytes collected at 24 hpi were analyzed for (D) NADPH, (E) R5P, (F–H) ROS and (I) viral load. Data are mean ± SD of three biological replicates. **p < 0.01, ***p < 0.001. ns, not significant.

Building on the identification of TKTL2-C1 fragment as the IE1-binding region, we next asked whether this fragment could act as a competitive inhibitor to disrupt IE1‑driven PPP activation and thereby suppress viral replication. To test this, recombinant GST-fusion proteins corresponding to TKTL2 fragments C1, C2, and C3 (TKTL2-C1/C2/C3-GST) were introduced into High Five cells co-expressing full-length TKTL2 and IE1. Co-IP assays showed that TKTL2-C1, but not C2 or C3, competitively reduced the interaction between IE1 and TKTL2 in a dose-dependent manner (Fig 8A and 8B).

We then evaluated the impact of this competitive inhibition on PPP activation and viral infection. None of the injected GST-fusion proteins showed cytotoxicity toward shrimp hemocytes (Fig 8C). Compared to shrimp injected with GST, TKTL2-C2-GST, or TKTL2-C3-GST, those receiving TKTL2-C1-GST exhibited significantly reduced levels of NADPH and R5P, elevated ROS, and lower viral loads in hemocytes (Fig 8D-8I). These results demonstrate that the C1 region of TKTL2 functions as a competitive inhibitor, blocking IE1-mediated PPP activation and thereby suppressing WSSV replication.

## Discussion

Viruses are highly skilled at manipulating host metabolism, rewiring cellular pathways to obtain biosynthetic precursors and energy required for their replication [25,26]. Among these, the pentose phosphate pathway (PPP) represents a key metabolic hub because it produces ribose-5-phosphate (R5P) for nucleotide synthesis and nicotinamide adenine dinucleotide phosphate (NADPH) for redox control and lipid biosynthesis [27]. Although the PPP has been shown to support the replication of several mammalian viruses, including HBV, HPV, and SARS-CoV-2 [9–11], its exploitation by invertebrate viruses has remained poorly understood. In this study, we reveal a previously unknown mechanism by which WSSV, a major pathogen in shrimp aquaculture, hijacks the host PPP. Specifically, WSSV uses its immediate-early protein IE1 to directly bind and activate the host enzyme transketolase-like protein 2 (TKTL2), thereby enhancing PPP flux, elevating NADPH and R5P levels, and reducing ROS. This reprogramming creates a favorable metabolic environment that promotes efficient viral replication.

We first demonstrated that WSSV infection strongly activates the PPP in *Penaeus vannamei*, as indicated by the upregulation of G6PD and TKTL2 expression and activity, coupled with elevated NADPH and R5P levels (Fig 1). Functional interference through RNAi-mediated silencing of G6PD or TKTL2, as well as pharmacological inhibition using 6-aminonicotinamide (6AN) or oxythiamine (OT), led to reduced viral replication and improved host survival (Fig 2). These data establish that the PPP is indispensable for WSSV infection. Similar findings have been reported in vertebrate systems, where inhibition of TKT restricts SARS-CoV-2 replication [11], and the DNA virus, infectious spleen and kidney necrosis virus (ISKNV) activates the PPP early in infection to support nucleotide synthesis in fish [28]. Our results are also consistent with previous multi-omics studies showing that WSSV induces a Warburg-like metabolic shift and temporally activates anabolic pathways, including the PPP [14,20].

A major finding of this study is that the viral immediate-early protein IE1 acts as a direct activator of the PPP. IE1 is known as a multifunctional regulator during WSSV infection, previously described as a transcriptional transactivator that interacts with host proteins such as the TATA-box binding protein (TBP) and thioredoxin [29–31]. It also modulates diverse host factors, including retinoblastoma protein (Rb) [32], signal transducer and activator of transcription (STAT) [33], c-Jun NH2-terminal kinase (JNK) [34], β-catenin [35], Chibby [36], prophenoloxidase (proPO) [24], integrin-α5 [37], and Src64B [38] to alter cellular signaling and facilitate viral replication and immune evasion. Here, we expand the functional repertoire of IE1 by showing that it directly binds to and activates the host metabolic enzyme TKTL2 (Figs 3, 4, 5). This mechanism differs from that of other viruses, as for example, while HBV upregulates G6PD transcriptionally and HPV promotes G6PD dimerization [9,10], WSSV employs its IE1 protein to post-translationally enhance TKTL2 activity through physical interaction. Such direct enzyme hijacking allows rapid and precise PPP activation, bypassing the slower transcriptional control. As an immediate-early gene product that is highly expressed throughout infection [39,40], IE1 likely initiates and maintains PPP activation from the earliest stages of viral replication.

Our molecular docking and pull-down analyses demonstrated that IE1 binds to the C-terminal region of TKTL2 (residues 500–555) and significantly enhances its enzymatic activity both *in vivo* and *in vitro* (Figs 4 and 5). To elucidate the underlying mechanism, we analyzed the domain architecture of TKTL2, which revealed that the IE1-binding site lies within a Ricin domain (residues 461–577), a substrate-binding module essential for sugar phosphate recognition and coordination in transketolases [41,42]. This precise localization suggests that IE1 allosterically optimizes the substrate-binding pocket, potentially enhancing affinity for donor substrates (e.g., xylulose-5-phosphate) or stabilizing a catalytically competent state to boost transketolation. By directly hijacking this regulatory domain, WSSV efficiently enhances flux through the non-oxidative PPP, elevating R5P and NADPH while reducing ROS (Fig 6). The functional importance of these metabolic changes was further supported by supplementation experiments, in which NADPH, R5P, or the ROS scavenger NAC restored viral replication following IE1 knockdown (Fig 7). Moreover, the TKTL2 C-terminal fragment (TKTL2-C1) acted as a competitive inhibitor that disrupted the IE1-TKTL2 interaction, suppressed PPP flux, and reduced viral replication (Fig 8), establishing a direct functional link between IE1-mediated PPP activation and successful WSSV propagation. Notably, shrimp TKTL2 shares > 60% sequence identity with orthologs from human, Drosophila, and zebrafish (S1 Fig), highlighting its evolutionary conservation. Expression profiling showed that TKTL2 is abundant in nerve tissue and hemocytes, the primary replication sites of WSSV [43], and expressed at lower levels in hepatopancreas and gills (S2 Fig). This expression pattern suggests that WSSV targets a pre-existing, metabolically active enzyme within its preferred replication niche to efficiently rewire host metabolism.

**Fig 9:**
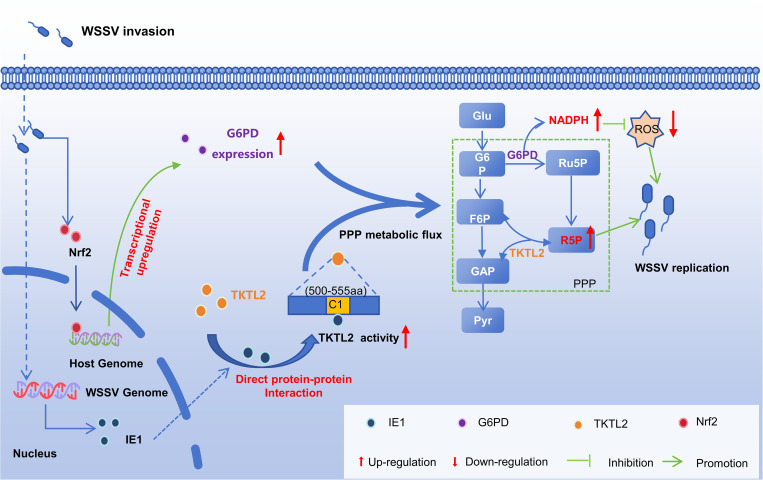
Schematic model of WSSV-mediated activation of the pentose phosphate pathway to promote viral replication. WSSV enhances host PPP activity through two complementary mechanisms. First, WSSV infection induces Nrf2 nuclear translocation, which upregulates antioxidant genes such as G6PD, increasing PPP flux and NADPH generation. Second, the viral protein IE1 directly interacts with TKTL2 and enhances its enzymatic activity. Together, these mechanisms promote NADPH and R5P production, maintaining redox homeostasis and providing nucleotide precursors to support efficient viral replication.

Within the broader context of WSSV-induced metabolic reprogramming, the IE1–TKTL2 axis integrates coherently with previously described pathways. WSSV infection induces a Warburg-like glycolytic phenotype and reprograms glutamine metabolism to fuel the TCA cycle and nucleotide synthesis [14,16,20]. Our results suggest that IE1-mediated activation of TKTL2 amplifies the non-oxidative branch of the PPP, supplying R5P to complement nucleotide precursors generated from glycolysis and glutaminolysis. At the same time, the increased NADPH pool supports reductive biosynthesis required for virion morphogenesis and counteracts oxidative stress, thereby maintaining redox balance and cellular viability during infection. This tight coordination of carbon and redox metabolism highlights the sophisticated strategy WSSV employs to optimize host resources for its replication cycle. Beyond its role in WSSV pathogenesis, the direct post‑translational hijacking of a metabolic enzyme may represent a distinct evolutionary strategy for rapid metabolic takeover. Unlike the more common transcriptional or signaling‑mediated rewiring employed by many large DNA viruses, direct enzyme activation could be particularly advantageous in invertebrate and aquatic hosts, where swift adaptation is critical. Future comparative studies of viral–host interactomes will help elucidate whether this mechanism is conserved among other large DNA viruses occupying similar ecological niches.

In summary, we identify a new mechanism of viral metabolic manipulation wherein WSSV, through its immediate‑early protein IE1, directly targets and activates TKTL2 to stimulate PPP flux and generate essential metabolites. Together with the previously described Nrf2–G6PD axis [23], this IE1–TKTL2 pathway forms a complementary two‑pronged strategy that enables WSSV to fully engage both branches of the PPP (Fig 9). This dual regulatory mode offers distinct advantages: transcriptional induction of G6PD sustains NADPH production for redox balance and biosynthesis throughout infection, whereas rapid post‑translational activation of TKTL2 by IE1 promptly boosts the non‑oxidative branch to accelerate R5P synthesis during critical stages such as viral genome replication. Such coordinated control not only provides metabolic flexibility for adapting to changing cellular conditions but also likely reflects an evolutionary adaptation to efficiently exploit host metabolism in a short‑lived invertebrate host. These findings advance our understanding of invertebrate virus–host interactions by demonstrating how viruses can directly activate host metabolic enzymes via protein–protein interactions to drive rapid metabolic remodeling. The IE1–TKTL2 interface thus represents a promising target for antiviral development, and its disruption could offer a novel strategy to control WSSV infection.

## Materials and methods

### Ethics statement

All experimental procedures involving animals were performed in compliance with institutional guidelines and were approved by the Animal Research and Ethics Committee of Shantou University, China (STU20251210001).

### Experimental animals

Healthy shrimp (*Penaeus vannamei*) with an average weight of 5–8 g were obtained from a commercial shrimp farm in Shantou, Guangdong Province, China. Prior to experimentation, shrimp were acclimated for 2–3 days in a recirculating aquaculture system (RAS) maintained at 25 °C and 5‰ salinity. During acclimation, shrimp were fed a commercial diet once daily.

### Antibodies and plasmids

Primary antibodies used in this study included rabbit polyclonal anti-G6PD (Beyotime, China, Cat. No. AF6945), mouse monoclonal anti-α-tubulin (Sigma-Aldrich, USA, Cat. No. T6074), rabbit polyclonal anti-FLAG (Beyotime, China, Cat. No. AF0036), mouse monoclonal anti-V5 (Sangon Biotech, China, Cat. No. D191104), mouse monoclonal anti-GST (Beyotime, China, Cat. No. AF0174), and mouse monoclonal anti-His (Beyotime, China, Cat. No. AF2876). In-house mouse polyclonal antibodies against TKTL2, IE1, and VP28 were generated by immunizing mice with purified recombinant proteins.

For Western blotting, secondary antibodies were goat anti-mouse IgG-HRP (ThermoFisher Scientific, USA, Cat. No. G21040) and goat anti-rabbit IgG-HRP (ThermoFisher Scientific, USA, Cat. No. G21234). For immunofluorescence assays, goat anti-rabbit IgG-Alexa Fluor 594 (Servicebio, China, Cat. No. GB28303) and goat anti-mouse IgG-Alexa Fluor 488 (Servicebio, China, Cat. No. GB25301) were used.

For eukaryotic expression, the full-length open reading frame (ORF) of *P. vannamei TKTL2* (GenBank accession number: XM_027380217) was cloned into the pIZ-V5-His vector with an N-terminal FLAG tag to generate pIZ-FLAG-TKTL2. Expression constructs for IE1 and EGFP (pIZ-V5-IE1 and pIZ-V5-EGFP) were obtained from a previous study [24]. For prokaryotic expression, wild-type TKTL2 and truncated mutants (TKTL2-C: 500–627 aa; TKTL2-C1: 500–555 aa; TKTL2-C2: 556–586 aa; TKTL2-C3: 587–627 aa) were cloned into pGEX-6p-1, yielding pGEX-TKTL2-GST, pGEX-TKTL2-C-GST, pGEX-TKTL2-C1/C2/C3-GST, and pGEX-TKTL2-C3-GST constructs. The full-length *IE1* ORF (GenBank accession number: AAL33073.1) was inserted into pET-28a to produce pET-28a-IE1-His. All plasmids were verified by DNA sequencing, and the primer sequences are provided in S1 Table.

### WSSV challenge and tissue collection

The WSSV China isolate (GenBank accession number: AF332093.3) was prepared from infected crayfish (*Procambarus clarkii*) and quantified by quantitative real-time PCR (qPCR) as described previously [44,45]. For viral challenge, shrimp were intramuscularly injected with 100 μL of WSSV suspension (5 × 10⁵ copies per shrimp). Control groups received sterile PBS (140 mM NaCl, 2.7 mM KCl, 10 mM Na₂HPO₄, 1.8 mM KH₂PO₄, pH 8.0).

Hemocyte and intestinal samples were collected from three biological replicates (each consisting of three shrimp) at 0, 6, 12, 24, 36, and 48 h post-infection for RNA and protein extraction. For hemocyte collection, hemolymph was withdrawn using a sterile 1 mL syringe preloaded with 600 μL of pre-chilled anticoagulant solution (0.45 M NaCl, 10 mM KCl, 10 mM EDTA, 10 mM HEPES, pH 7.45). Hemocytes were pelleted by centrifugation at 800 × g for 10 min at 4 °C. Intestinal tissues were dissected aseptically, placed in enzyme-free microcentrifuge tubes, snap-frozen in liquid nitrogen, and stored at −80 °C until analysis.

### RNA isolation, cDNA synthesis, and quantitative PCR (qPCR) analysis

Total RNA was extracted from hemocyte and intestinal tissues using the Fastagen Total RNA Rapid Extraction Kit (Fastagen, China, Cat. No. 220011) and the TransZol Up Plus RNA Kit (TransGen Biotech, China, Cat. No. ER501), respectively. Complementary DNA (cDNA) was synthesized from 1 µg of total RNA using the Evo M-MLV Reverse Transcription Kit (Accurate Biology, China, Cat. No. AG11705).

The relative mRNA expression levels of TKTL2, G6PD, and IE1 in hemocytes and intestinal tissues following WSSV infection were quantified by qPCR. Each 20 µL reaction contained 10 µL of 2 × SYBR Green Pro Taq HS Premix (Accurate Biology, China, Cat. No. AG11701), 1 µL each of forward and reverse primers, 1 µL of cDNA template, and 7 µL of Milli-Q water. Amplification was performed on a LightCycler 480 system (Roche, Switzerland) under the following cycling conditions: 95 °C for 30 s, followed by 40 cycles of 95 °C for 5 s and 60 °C for 30 s. Expression levels were normalized to elongation factor 1-α (EF-1α) and calculated using the 2^−ΔΔCT^ method. Primer sequences are listed in S1 Table.

### Protein extraction and Western blot analysis

For protein extraction from hemocytes, cells were resuspended in 150 μL of ice-cold IP/Western blot lysis buffer (50 mM Tris-HCl, 150 mM NaCl, 2 mM EDTA-2Na, 1% Triton X-100, pH 8.0) supplemented with 1 × PMSF (Beyotime, China, Cat. No. ST505) and 1 × protease inhibitor cocktail (MCE, USA, Cat. No. HY-K0010). The mixture was gently rotated at 4 °C for 20 min and centrifuged at 16,000 × g for 10 min at 4 °C. The resulting supernatant was mixed with protein loading buffer (50 mM Tris-HCl, 2% SDS, 0.1% BPB, 10% glycerin, 1% β-mercaptoethanol, pH6.8) and boiled for 10 min.

For intestinal tissues, samples were homogenized for 1 min at 70 Hz in IP/Western blot lysis buffer using a tissue homogenizer (Servicebio, China), and incubated at 4 °C with rotation for 20 min, and centrifuged at 16,000 × g for 10 min at 4 °C. Supernatants were mixed with protein loading buffer and boiled for 10 min.

Protein samples were separated by 12% SDS-PAGE and transferred onto polyvinylidene difluoride (PVDF) membranes (Millipore, USA, Cat. No. IPVH00010). Membranes were blocked with 5% non-fat milk in TBST (20mM Tris, 150 mM NaCl, 0.1% Tween, pH7.4) for 1 h at room temperature and incubated overnight at 4 °C with primary antibodies, i.e., anti-G6PD, anti-TKTL2, anti-VP28, or anti-α-tubulin. After washing, membranes were incubated with HRP-conjugated secondary antibodies for 1 h at room temperature. Protein bands were visualized using an Amersham Imager 600 system (GE Healthcare, USA).

### RNA interference (RNAi) assay

Double-stranded RNAs (dsRNAs) targeting *P. vannamei* G6PD, TKTL2, and the control EGFP were synthesized *in vitro* using the HiScribe T7 Quick High Yield RNA Synthesis Kit (New England Biolabs, USA; Cat. No. E2050S), following the manufacturer’s instructions. The primers used for dsRNA synthesis are listed in S1 Table. Each shrimp was intramuscularly injected with 10 μg of the corresponding dsRNA, with dsRNA targeting EGFP (dsEGFP) used as a non-specific silencing control. At 48 h post-dsRNA injection, shrimp were challenged with 100 μL of WSSV suspension (5 × 10⁵ copies per shrimp). Hemocytes were collected at 24 and 48 h post-infection. A subset of samples was processed for RNA and protein extraction to assess the knockdown efficiency of G6PD and TKTL2 and the expression of viral genes (IE1 and VP28) by qPCR and Western blotting. Remaining hemocyte samples were used for DNA extraction with the Marine Animal DNA Extraction Kit (TIANGEN, China; Cat. No. DP324) for quantification of viral load as previously described [45].

### Shrimp survival rate assay

Healthy shrimp of similar size were randomly assigned to three treatment groups (dsEGFP, dsTKTL2, and dsG6PD) for RNAi experiments. Each shrimp was intramuscularly injected with 10 μg of the corresponding dsRNA. After 48 h, each group was subdivided into two subgroups (n = 40 per subgroup). One subgroup was challenged with 100 μL of WSSV suspension (5 × 10⁵ copies per shrimp), while the other received 100 μL of sterile PBS as a control. Shrimp survival was monitored every 12 h post-infection. Survival curves were generated using GraphPad Prism, and statistical differences between groups were analyzed using the log-rank test.

### Inhibitor treatment assay

The G6PD inhibitor 6-aminonicotinamide (6AN; MCE, USA; Cat. No. HY-W010342) and the TKT inhibitor oxythiamine (OT; MCE, USA; Cat. No. HY-107430) were dissolved in dimethyl sulfoxide (DMSO). For inhibition assays, shrimp were intramuscularly injected with 2 μM OT or 20 μM 6AN, while control animals received an equivalent volume of DMSO. At 2 h post-injection, shrimp were challenged with 100 μL of WSSV suspension (5 × 10⁵ copies per shrimp). Hemocytes were collected at 24 h post-infection, and total DNA and protein were extracted to assess WSSV load and viral gene expression (IE1 and VP28) using qPCR and Western blot, respectively.

To evaluate potential cytotoxicity of the inhibitors, shrimp were treated with OT or 6AN as described above, and hemocytes were collected 24 h post-treatment. Cell viability was assessed using the CCK-8 kit (Beyotime, China; Cat. No. C0037) according to the manufacturer’s protocol.

### Molecular docking analysis

The three-dimensional structures of IE1 and TKTL2 were modeled using the SWISS-MODEL server based on homologous templates. The crystal structure of the SR1 domain of human sacsin (PDB: 5V44; 25.64% sequence similarity to IE1) was used as the template for IE1, whereas the human transketolase variant E160Q (PDB: 6HA3; 61.45% sequence similarity to *P. vannamei* TKTL2) served as the template for TKTL2. Protein-protein docking was performed using the HDOCK server, which employs a hybrid algorithm for predicting complex formation. In the docking setup, TKTL2 and IE1 were designated as receptor and ligand, respectively. The resulting complex structures and interface residues were analyzed using the MOE contact module, and all structural visualizations were generated using PyMOL.

### GST pull-down assay

Prokaryotic expression plasmids encoding wild-type TKTL2 and its truncated variants (pGEX-TKTL2-GST, and pGEX-TKTL2-C/C1/C2/C3-GST), as well as the IE1 expression plasmid pET-28a-IE1-His, were transformed into *Escherichia coli* BL21 (DE3) cells. Protein expression was induced with 1 mM IPTG at 16 °C. After induction, bacterial cells were harvested by centrifugation and lysed by ultrasonication on ice for 15 min in PBS. The supernatants were incubated with glutathione magnetic beads (Beaver, China, Cat. No. 70601) or Ni-NTA agarose (Cytiva, Sweden, Cat. No. 17531801) to purify the respective GST- and His-tagged following the manufacturers’ protocols.

For GST pull-down assays, purified GST-tagged proteins were immobilized on glutathione magnetic beads at 4 °C and incubated overnight with purified His-tagged IE1 protein at a 1:1 volume ratio. Beads were washed three times with PBS to remove nonspecific proteins, and the bound complexes were eluted by boiling in protein loading buffer. Eluted samples were separated by 12% SDS-PAGE, transferred to PVDF membranes, and analyzed by Western blot using anti-His antibody to detect interactions between IE1 and TKTL2 or its truncated mutants.

### Co-immunoprecipitation (Co-IP) assay

High Five cells were seeded in 6-well plates at a density of 1 × 10⁶ cells per well and allowed to adhere before transfection. For co‑transfection, each well received 1 μg pIZ‑FLAG‑TKTL2 and 1 μg of either pIZ‑V5‑IE1 or pIZ‑V5‑EGFP plasmid, using 4 μL of FuGENE HD transfection reagent (Promega, USA, Cat. No. E2311) according to the manufacturer’s protocol. After 48 h for transfection, cells were collected by centrifugation at 500 × g for 5 min at 4 °C, washed once with PBS, and resuspended in 450 μL of IP-Western blot lysis buffer. Lysates were incubated on ice for 20 min and centrifuged at 16,000 × g for 10 min at 4 °C. A small aliquot of the supernatant was retained as an input control, while the remaining supernatant was incubated overnight at 4 °C with anti‑FLAG M2 magnetic beads (Sigma, USA, Cat. No. M8823) or with protein A/G magnetic beads (MCE, USA, Cat. No. HY-K0202) pre-bound with an anti-V5 antibody. Beads were washed three times with lysis buffer to remove non-specific proteins, resuspended in 30 μL of PBS, and mixed with protein loading buffer. Immunoprecipitated proteins were eluted by boiling for 10 min and analyzed by Western blot using anti-FLAG and anti-V5 antibodies.

For competitive co‑IP assays, cells co‑transfected with pIZ‑FLAG‑TKTL2 and pIZ‑V5‑IE1 were harvested 48 h post‑transfection. Cell lysates were mixed with 100 μM of the indicated recombinant GST‑fusion proteins (TKTL2‑C1/C2/C3‑GST) or with increasing concentrations of TKTL2‑C1‑GST (0, 1, 10, and 100 μM). The mixtures were incubated overnight at 4 °C with anti‑FLAG M2 magnetic beads. After incubation, the beads were washed three times with lysis buffer to remove nonspecifically bound proteins, resuspended in 30 μL of PBS, and mixed with protein loading buffer for denaturation. Samples were boiled for 15 min, and the eluted proteins were analyzed by Western blot using anti‑FLAG and anti‑V5 antibodies.

### Endogenous Co-IP assay

Shrimp were injected with 100 μL of WSSV suspension (5 × 10⁵ copies per shrimp), and hemocytes were collected 24 h post-infection. Cells were washed three times with PBS and lysed in 300 μL of IP-Western blot lysis buffer for 20 min at 4 °C. Lysates were centrifuged at 16,000 × g for 10 min at 4 °C, and the supernatant was divided into equal portions. One portion was incubated overnight at 4 °C with anti-IE1 antibody, and the other with control Mouse IgG (Beyotime, China, Cat. No. A7028). After incubation, protein A/G magnetic beads were added and incubated for an additional 6 h at 4 °C. The beads were then washed three times to remove nonspecific binding, resuspended in 30 μL of PBS, and mixed with protein loading buffer. Samples were boiled for 15 min, and eluted proteins were subjected to Western blot analysis using anti-TKTL2 and anti-IE1 antibodies.

### Immunofluorescence assay

High Five cells were seeded in 24-well plates at a density of 1 × 10⁵ cells per well and co-transfected with 1 μg each of FLAG-tagged TKTL2 and V5-tagged IE1 expression plasmids using FuGENE HD transfection reagent. At 48 h post-transfection, cells were transferred to confocal dishes and allowed to adhere for 4 h at 28 °C. After washing three times with PBS, cells were fixed with 4% paraformaldehyde for 10 min at 4 °C, followed by another three washes with PBS. Cells were blocked with 3% BSA in PBS for 1 h at room temperature and incubated overnight at 4 °C with rabbit anti-FLAG (1:200) and mouse anti-V5 (1:200) primary antibodies. After washing, cells were incubated for 1 h at room temperature with secondary antibodies: goat anti-mouse IgG-Alexa Fluor 488 (1:200) and goat anti-rabbit IgG-Alexa Fluor 594 (1:200). Nuclei were counterstained with Hoechst 33342 (Beyotime, China, Cat. No. C1022) for 15 min. Images were captured using a Zeiss LSM 800 confocal microscope (Carl Zeiss, Germany) and processed with ZEN (Blue edition) software.

### Measurements of TKT and G6PD enzymatic activities in cells

TKT activity was determined using the TKT Activity Assay Kit (Abcam, UK, Cat. No. ab273310) following the manufacturers’ instructions. Briefly, cell lysates were prepared from 5 × 10⁶ cells and quantified using a BCA Assay Kit (GenStar, China, Cat. No. E162). Each reaction contained 1 µg of total protein, and fluorescence (excitation/emission = 535/587 nm) was recorded immediately and every 5 min for 45 min at 37 °C. Enzyme activity was calculated according to the formula: TKT activity = [ΔM/(Δt × P)] × D, where ΔM represents the G3P concentration interpolated from the standard curve (pmol), Δt is the reaction time (min), D is the sample dilution factor, and P is the amount of protein used (µg). One unit (U) of TKT activity was defined as the amount of enzyme that generates 1 μmol of G3P per minute under the assay conditions.

G6PD activity was measured using the G6PD Activity Assay Kit (Solarbio, China, Cat. No. BC0260) according to the manufacturer’s protocol. Briefly, approximately 1 × 10⁶ cells were homogenized in lysis buffer and centrifuged at 8,000 × g for 10 min at 4 °C to collect the supernatant. A 10 μL aliquot of the supernatant was then mixed with the reaction solution. The absorbance at 340 nm was recorded immediately (A1) and after a 5-min incubation at 37 °C (A2). The total protein concentration in the supernatant was determined in parallel using a BCA assay kit. G6PD activity was calculated using the equation: G6PD activity (U/mg protein) = 1072 × (A2 – A1) ÷ Cpr × F (Cpr = Sample protein concentration; F = Dilution factor). One unit of enzyme activity was defined as the amount of G6PD required to generate 1 nmol of NADPH per minute under the assay conditions.

### *In vitro* TKT enzymatic activity assay

Recombinant TKTL2-GST and IE1-His proteins were expressed and purified as described above. Protein concentrations were determined using a BCA assay and adjusted to 1 μM. To examine whether IE1 affects TKTL2 enzymatic activity, 200 µL aliquots of TKTL2-GST and IE1-His were mixed and incubated at 4°C for 6 h. After incubation, the transketolase activity of TKTL2 was measured using the TKT Activity Assay Kit. Control reactions containing TKTL2-GST alone, IE1-His alone, or GST alone were included to determine baseline activity and assess the specificity of the interaction.

### Measurements of NADPH and R5P

Cellular NADPH levels were quantified using the NADP ⁺ /NADPH Assay Kit (Beyotime, China, Cat. No. S0179) per the manufacturer’s protocol. Briefly, approximately 1 x 10⁶ cells were collected after WSSV infection or IE1/TKTL2 overexpression, washed with PBS, and lysed. The lysates were centrifuged, and the supernatants were incubated at 60°C for 30 min to selectively degrade NADP ⁺ . Absorbance at 450 nm was measured, and NADPH concentrations were calculated using a standard curve.

For ribose-5-phosphate (R5P) measurement, metabolites were extracted from 1 × 10⁷ cells by snap-freezing in ice-cold 80% (v/v) methanol. Samples were sonicated and centrifuged at 14,000 × g for 10 min at 4 °C. The resulting supernatants were lyophilized and reconstituted in 200 μL of 50% methanol (v/v) for analysis. Metabolite separation was performed using a Thermo Fisher LC-MS system equipped with a Hypersil GOLD column (1.9 μm, 50 × 2.1 mm; PN: 25002–052130). The mobile phase consisted of (A) methanol and (B) 15 mM ammonium acetate, with a flow rate of 0.16 mL/min and an injection volume of 5 μL. Detection was conducted in negative ionization mode under the following parameters: ion spray voltage (negative), sheath gas 40 Arb, auxiliary gas 10 Arb, ion transfer tube temperature 300 °C, and vaporizer temperature 300 °C. R5P was quantified by monitoring the precursor ion at m/z 228.848 and the product ion at m/z 97.04, with concentrations determined from a standard curve.

### ROS analysis

Cellular ROS levels were determined using the ROS Detection Kit (AAT Bioquest, USA; Cat. No. 15204) following the manufacturer’s instructions. Briefly, approximately 1 x 10⁷ cells were collected, washed with PBS, and stained according to the kit protocol. Fluorescence intensity (Ex/Em = 488/525 nm) was measured using a microplate reader, and ROS levels were further verified by flow cytometry (Accuri C6 Plus, BD, USA).

### Exogenous metabolite supplementation and rescue assay

D-ribose-5-phosphate (R5P; LUBEX, Cat. No. S28617) and NADPH (Beyotime, China, Cat. No. ST360) were dissolved in DEPC-treated H_2_O. The cytotoxicity of these metabolites at 10 μM was evaluated using the Cell Counting Kit-8 (Beyotime, China, Cat. No. C0037) according to the manufacturer’s instructions, and no significant toxicity was observed. For metabolite supplementation assays, shrimp were intramuscularly injected with 10 μM R5P or NADPH, while control shrimp received an equal volume of DEPC-H₂O. Two hours post-injection, shrimp were challenged with WSSV (5 × 10⁵ copies per shrimp). Hemocytes were collected at 24 h post-infection (hpi) for quantification of viral gene expression and viral load.

For the rescue assays, shrimp were pretreated with dsIE1 and, after 12 h, co-injected with WSSV (5 × 10⁵ copies per shrimp) together with 10 μM NADPH, 10 μM R5P, 10 μM of the ROS scavenger N-acetylcysteine (NAC; MCE, Cat. No. HY-B0215), or an equal volume of DEPC-H₂O as a control. Hemocytes were collected at 24 hpi for subsequent analysis of viral gene expression and viral load.

### Protein‑blocking assay

Recombinant GST or GST‑fusion proteins (TKTL2‑C1/C2/C3‑GST) were expressed and purified as described above. To remove endotoxin contamination, proteins were washed with 0.1% Triton X‑114 prior to final elution. For the blocking assay, 10 μM of each GST or GST‑tagged protein was pre‑incubated with WSSV (5 × 10⁵ copies) and then injected into shrimp. At 24 h post‑infection, hemocytes were collected for quantification of NADPH, R5P, ROS and WSSV load as described above. Cytotoxicity of the recombinant proteins toward shrimp hemocytes was evaluated using a CCK‑8 kit according to the manufacturer’s protocol.

### Statistical analysis

All experiments were performed with at least three independent biological replicates. Data are presented as mean ± standard deviation (SD). Statistical significance between two groups was determined using a two-tailed Student’s t-test. Graphing was conducted using GraphPad Prism 10 software (GraphPad Software, USA).

## Supporting information

S1 Fig Multiple sequence alignment of transketolase-like 2 (TKTL2) from *Penaeus vannamei* and other species. The aligned sequences include *P. vannamei* (*Pv*TKTL2, XM_027380217), Homo sapiens (HsTKTL2, NP_115512.3), Danio rerio (*Dr*TKTL2, NP_932336.3), Mus musculus (*Mm*TKTL2, NP_001258503.1), and Drosophila melanogaster (*Dm*TKTL2, NP_649812.2). The sequence identity between *Pv*TKTL2 and each ortholog is indicated at the end of the alignment.
(DOCX)

S2 Fig Tissue distribution analysis of TKTL2 in *Penaeus vannamei.* The relative expression of TKTL2 mRNA across various shrimp tissues was quantified by qPCR, with normalization to the EF1α reference gene.
(DOCX)

S1 Table Primers used in this study. (DOCX)

S1 Data Raw data in this study. (DOCX)
