# Supplementary material for: White spot syndrome virus IE1 protein hijacks the host pentose phosphate pathway to fuel viral replication
Source: PLoS Pathog. 2026 Jan 27;22(1):e1013913. doi: 10.1371/journal.ppat.1013913 (PMC12858063; doi:10.1371/journal.ppat.1013913)
Supplement: S1 Fig — The aligned sequences include P. vannamei (PvTKTL2, XM_027380217), Homo sapiens (HsTKTL2, NP_115512.3), Danio rerio (DrTKTL2, NP_932336.3), Mus musculus (MmTKTL2, NP_001258503.1), and Drosophila melanogaster (DmTKTL2, NP_649812.2). The sequence identity between PvTKTL2 and each ortholog is indicated at the end of the alignment. (DOCX) [file ppat.1013913.s001.docx]

**S1 Fig. Multiple sequence alignment of transketolase-like 2 (TKTL2) from**

***Penaeus vannamei* and other species.** The aligned sequences include: *P. vannamei* (*Pv*TKTL2, XM_027380217), *Homo sapiens* (*Hs*TKTL2, NP_115512.3), *Danio rerio* (*Dr*TKTL2, NP_932336.3), *Mus musculus* (*Mm*TKTL2, NP_001258503.1), and *Drosophila melanogaster* (*Dm*TKTL2, NP_649812.2). The sequence identity between *Pv*TKTL2 and each ortholog is indicated at the end of the alignment.
