# Supplementary material for: White spot syndrome virus IE1 protein hijacks the host pentose phosphate pathway to fuel viral replication
Source: PLoS Pathog. 2026 Jan 27;22(1):e1013913. doi: 10.1371/journal.ppat.1013913 (PMC12858063; doi:10.1371/journal.ppat.1013913)
Supplement: S2 Fig — The relative expression of TKTL2 mRNA across various shrimp tissues was quantified by qPCR, with normalization to the EF1α reference gene. (DOCX) [file ppat.1013913.s002.docx]

**S2 Fig. Tissue distribution analysis of TKTL2 in *Penaeus vannamei***. The relative expression of

*TKTL2* mRNA across various shrimp tissues was quantified by qPCR, with normalization to the

*EF1α* reference gene.
