# Supplementary material for: White spot syndrome virus IE1 protein hijacks the host pentose phosphate pathway to fuel viral replication
Source: PLoS Pathog. 2026 Jan 27;22(1):e1013913. doi: 10.1371/journal.ppat.1013913 (PMC12858063; doi:10.1371/journal.ppat.1013913)
Supplement: S1 Table — (DOCX) [file ppat.1013913.s003.docx]

**S1 Table Primers used in this study**

| **Primer name** | **Sequence (5’-3’)** |
| --- | --- |
| **For plasmid construction** | |
| Eukaryon-TKTL2-F(KpnI) | GGGGTACCATGGATTACAAGGACGACGATGACAAGATGTCTGAGTACCACAAACCCGAT |
| Eukaryon-TKTL2-R(XhoI) | CCCTCGAGTTAAAGGCTAGATATCTCTTTGGCAG |
| Prokaryote-TKTL2-F(BamHⅠ) | CGGGATCCGCGATGTCTGAGTACCACAAACCCGAT |
| Prokaryote-TKTL2-R(XhoI) | CCCTCGAGTTAAAGGCTAGATATCTCTTTGGCAG |
| TKTL2-C-F(BamHⅠ) | CGGGATCCGCGATGAAGTCGTCAGGCAGTGA |
| TKTL2-C-R(XhoI) | CCCTCGAGTTAAAGGCTAGATATCTCTTTGGCAG |
| TKTL2-C1-F(BamHⅠ) | CGGGATCCGCGATGAAGTCGTCAGGCAGT |
| TKTL2-C1-R(XhoI) | CCCTCGAGTCAACGAGCATTTTTGATAA |
| TKTL2-C2-F(BamHⅠ) | CGGGATCCGCGATGGAGTGTGGTGGCCGT |
| TKTL2-C2-R(XhoI) | CCCTCGAGTCAGTCACGCTCCATTGC |
| TKTL2-C3-F(BamHⅠ) | CGGGATCCGCGATGATCATCATGAAACACCTTGC |
| TKTL2-C3-F(BamHⅠ) | CCCTCGAGTTAAAGGCTAGATATCTCTTTGGCAG |
| **For qRT-PCR assay** | |
| q-IE1-F | GCACAACAACAGACCCTACCC |
| q-IE1-R | GAAATACGACATAGCACCTCCAC |
| q-VP28-F | AAACCTCCGCATTCCTGTGA |
| q-VP28-R | TCCGCATCTTCTTCCTTCAT |
| q-EF-1α-F | TATGCTCCTTTTGGACGTTTTGC |
| q-EF-1α-R | CCTTTTCTGCGGCCTTGGTAG |
| TKTL2_qPCR_F | TCGGCCAGTAACTCAGGG |
| TKTL2_qPCR_R | CAGCATAGAGGATGGGAGC |
| G6PD-qPCR-F | AGGAGCGGTACGAGCAGTTC |
| G6PD-qPCR-R | GCAGCACGCCTTCAGGTT |
| **For RNAi assay** | |
| dsTKLTL2-T7F | GGATCCTAATACGACTCACTATAGGAGGGACATCGCAAACAAG |
| dsTKLTL2-R | CTGCAAAGCTCAAAGCC |
| dsTKLTL2-F | AGGGACATCGCAAACAAG |
| dsTKLTL2-T7R | GGATCCTAATACGACTCACTATAGGCTGCAAAGCTCAAAGCC |
| dsG6PD-T7F | GGATCCTAATACGACTCACTATAGGCGCCACCATCAACAACGA |
| dsG6PD-R | TCTGCCAGCTCATCACTCC |
| dsG6PD-F | CGCCACCATCAACAACGA |
| dsG6PD-T7R | GGATCCTAATACGACTCACTATAGGTCTGCCAGCTCATCACTCCT |
