# Supplementary material for: White spot syndrome virus IE1 protein hijacks the host pentose phosphate pathway to fuel viral replication
Source: PLoS Pathog. 2026 Jan 27;22(1):e1013913. doi: 10.1371/journal.ppat.1013913 (PMC12858063; doi:10.1371/journal.ppat.1013913)
Supplement: S1 Data — (DOCX) [file ppat.1013913.s004.docx]

Fig.1

| **IE1** | **PBS** | | | **Mean±SD** | **WSSV** | | | **Mean±SD** |
| --- | --- | --- | --- | --- | --- | --- | --- | --- |
| 0h | 0.854015011 | 0.887816443 | 1.31889831 | 1.020243254±0.259194455 | 0.854015011 | 0.887816443 | 1.31889831 | 1.020243254±0.259194455 |
| 6h | 0.940391454 | 0.813003424 | 0.747079321 | 0.8334914±0.09827112 | 3.551265771 | 3.341123552 | 3.899619423 | 3.597336249±0.28208381 |
| 12h | 1.289072435 | 0.755934602 | 0.918488354 | 0.987831797±0.273249652 | 7.151933555 | 11.25374547 | 11.71539209 | 10.04035704±2.512075248 |
| 24h | 0.629087819 | 0.403973716 | 0.925518243 | 0.652859926±0.261583655 | 452.3618121 | 628.3102621 | 560.4078418 | 547.0266386±88.73419135 |
| 36h | 0.629960525 | 0.649919677 | 1.464424011 | 0.914768071±0.476120606 | 856.5141874 | 823.9045122 | 1289.265195 | 989.8946315±259.7747069 |
| 48h | 1.042706649 | 1.268684494 | 1.191132028 | 1.167507724±0.114826292 | 8262.332094 | 7133.198143 | 5745.964377 | 7047.164871±1260.388002 |

Fig.1A WSSV challenge（The mRNA levels of the viral gene IE1 in Hemocytes）

Fig.1B WSSV challenge（The mRNA levels of the viral gene IE1 in Intestine）

| **IE1** | **PBS** | | | **Mean±SD** | **WSSV** | | | **Mean±SD** |
| --- | --- | --- | --- | --- | --- | --- | --- | --- |
| 0h | 0.591178893 | 1.246313202 | 1.357231357 | 1.064907817±0.413992789 | 0.591178893 | 1.246313202 | 1.357231357 | 1.064907817±0.413992789 |
| 6h | 1.110082321 | 2.356534278 | 1.711980972 | 1.72619919±0.623347607 | 2.518677954 | 2.536196745 | 2.382814538 | 2.479229746±0.083956216 |
| 12h | 1.624129496 | 1.273383227 | 1.782209166 | 1.559907296±0.260421442 | 22.74798206 | 31.13934443 | 28.14226743 | 27.34319797±4.252366941 |
| 24h | 1.564462054 | 1.358172444 | 1.846337978 | 1.589657492±0.245056126 | 64.11840652 | 72.23702968 | 111.8690132 | 82.74148313±25.54971167 |
| 36h | 1.00742097 | 1.119354233 | 1.088745433 | 1.071840212±0.057849838 | 249.9796192 | 158.2066757 | 245.1747619 | 217.7870189±51.65398957 |
| 48h | 0.420934209 | 0.396575283 | 0.482856588 | 0.43345536±0.044482584 | 372.6472148 | 433.1331149 | 262.9540062 | 356.2447786±86.26709798 |

| **G6PD** | **PBS** | | | **Mean±SD** | **WSSV** | | | **Mean±SD** |
| --- | --- | --- | --- | --- | --- | --- | --- | --- |
| 0h | 0.722799126 | 0.959042508 | 1.442595338 | 1.041478991±0.366910721 | 0.722799126 | 0.959042508 | 1.442595338 | 1.041478991±0.366910721 |
| 6h | 1.161240397 | 0.987372704 | 0.806455407 | 0.985022836±0.177404168 | 1.767446648 | 2.788842899 | 1.760111308 | 2.105466952±0.591832295 |
| 12h | 0.921890091 | 0.704009434 | 1.690753883 | 1.105551136±0.518376973 | 2.103317533 | 2.93969538 | 4.677269853 | 3.240094255±1.313006949 |
| 24h | 0.980552422 | 1.744322341 | 1.175004813 | 1.299959859±0.396921207 | 5.718618511 | 6.541704839 | 6.76301553 | 6.34111296±0.550335452 |
| 36h | 0.56006519 | 0.649169294 | 1.193060056 | 0.800764846±0.342646368 | 13.98362665 | 11.49286569 | 13.78155397 | 13.08601544±1.383402664 |
| 48h | 0.558127511 | 1.225751619 | 0.792600986 | 0.858826705±0.338703221 | 42.6852779 | 37.94049685 | 30.30883862 | 36.97820446±6.244082581 |
|  |  |  |  |  |  |  |  |  |

Fig.1C WSSV challenge（The mRNA levels of the G6PD in Hemocytes）

Fig.1D WSSV challenge（The mRNA levels of the G6PD in Intestine）

| **G6PD** | **PBS** | | | **Mean±SD** | **WSSV** | | | **Mean±SD** |
| --- | --- | --- | --- | --- | --- | --- | --- | --- |
| 0h | 1.371733289 | 0.648419777 | 1.124278924 | 1.048143997±0.367618014 | 1.371733289 | 0.648419777 | 1.124278924 | 1.048143997±0.367618014 |
| 6h | 0.944747041 | 0.785128119 | 1.190031696 | 0.973302285±0.203956559 | 1.076240125 | 1.110338834 | 1.374588696 | 1.187055885±0.163300633 |
| 12h | 0.866937564 | 1.157490217 | 1.002081605 | 1.008836462±0.145394058 | 1.42899414 | 1.489677463 | 1.279872414 | 1.399514672±0.107964434 |
| 24h | 1.049716684 | 1.053361036 | 0.655196702 | 0.919424807±0.228835507 | 3.721798631 | 2.751083636 | 2.034959384 | 2.835947217±0.846615634 |
| 36h | 0.810003474 | 1.191682575 | 0.787307977 | 0.929664675±0.227197725 | 5.00371632 | 4.475348404 | 5.212588597 | 4.897217774±0.379983198 |
| 48h | 0.878430468 | 1.105730653 | 0.971307496 | 0.985156206±0.11428116 | 9.9176616 | 11.05785765 | 8.016652841 | 9.664057364±1.536381464 |

| TKTL2 | **PBS** | | | **Mean±SD** | **WSSV** | | | **Mean±SD** |
| --- | --- | --- | --- | --- | --- | --- | --- | --- |
| 0h | 0.709561678 | 0.908148418 | 1.551861709 | 1.056523935±0.440316711 | 0.709561678 | 0.908148418 | 1.551861709 | 1.056523935±0.440316711 |
| 6h | 1.31494276 | 1.310393404 | 0.982820599 | 1.202718921±0.190451118 | 1.332374825 | 1.901318202 | 1.802500925 | 1.678731318±0.303995606 |
| 12h | 1.035264924 | 0.581560021 | 0.911301281 | 0.842708742±0.234501039 | 4.39541687 | 4.242750965 | 6.337878487 | 4.992015441±1.168048468 |
| 24h | 0.845572287 | 0.814507563 | 1.199971382 | 0.953350411±0.214144068 | 6.666825657 | 10.62212127 | 8.907372946 | 8.732106623±1.983464029 |
| 36h | 0.378404198 | 0.637280314 | 0.843815796 | 0.619833436±0.233195806 | 9.36968145 | 7.155239214 | 12.99603834 | 9.840319669±2.948704607 |
| 48h | 1.241427492 | 0.980099415 | 0.897510051 | 1.039678986±0.179532993 | 29.222344 | 24.00062383 | 22.84806367 | 25.3570105±3.396719155 |
|  |  |  |  |  |  |  |  |  |

Fig.1E WSSV challenge（The mRNA levels of the TKTL2 in Hemocytes）

Fig.1F WSSV challenge（The mRNA levels of the TKTL2 in Intestine）

| TKTL2 | **PBS** | | | **Mean±SD** | **WSSV** | | | **Mean±SD** |
| --- | --- | --- | --- | --- | --- | --- | --- | --- |
| 0h | 0.897717443 | 1.328992841 | 0.838180773 | 1.021630352±0.267843104 | 0.897717443 | 1.328992841 | 0.838180773 | 1.021630352±0.267843104 |
| 6h | 0.746044366 | 1.320727959 | 1.206922746 | 1.09123169±0.304308434 | 1.602509971 | 1.721102874 | 1.768263571 | 1.697292139±0.085403614 |
| 12h | 0.758033406 | 1.30616154 | 1.096064982 | 1.053419976±0.276541245 | 1.824287782 | 1.951161685 | 1.835703853 | 1.87038444±0.070187635 |
| 24h | 1.230575635 | 1.264295179 | 0.711203009 | 1.068691274±0.310052652 | 3.992613259 | 3.108747265 | 3.106593193 | 3.402651239±0.510923232 |
| 36h | 1.324394875 | 1.228019384 | 0.775572381 | 1.10932888±0.293030902 | 3.451762813 | 3.681601135 | 4.034341843 | 3.722568597±0.293442211 |
| 48h | 1.497268889 | 0.870148376 | 1.441262713 | 1.269559993±0.347032279 | 7.718568639 | 11.01450941 | 11.50615044 | 10.07974283±2.059559515 |

Fig.1I Enzymatic activity of G6PD

| **G6PD** | **PBS** | | | **Mean±SD** | **WSSV** | | | **Mean±SD** |
| --- | --- | --- | --- | --- | --- | --- | --- | --- |
| 24h | 25.04004271 | 28.61719167 | 34.34063001 | 29.33262146±4.691386882 | 23.60918313 | 28.61719167 | 34.34063001 | 28.85566827±5.369696579 |

Fig.1J Enzymatic activity of TKT

| **TKT** | **PBS** | | | **Mean±SD** | **WSSV** | | | **Mean±SD** |
| --- | --- | --- | --- | --- | --- | --- | --- | --- |
| 24h | 87.31604857 | 62.75917731 | 58.94467664 | 69.67330084±15.39764639 | 182.0472154 | 218.0295137 | 144.5791963 | 181.5519751±36.72766299 |

Fig.1J NADPH levels

| **NADPH** | **PBS** | | | **Mean±SD** | **WSSV** | | | **Mean±SD** |
| --- | --- | --- | --- | --- | --- | --- | --- | --- |
| 24h | 2.287659666 | 2.287659666 | 2.405106479 | 2.326808604±0.067807949 | 3.344680985 | 3.360000134 | 3.227234172 | 3.31063843±0.072635197 |

Fig.1L R5P levels

| **R5P** | **PBS** | | | **Mean±SD** | **WSSV** | | | **Mean±SD** |
| --- | --- | --- | --- | --- | --- | --- | --- | --- |
| 24h | 0.989795918 | 1.114285714 | 1.142857143 | 1.082312925±0.081385681 | 2.128571429 | 1.957142857 | 2.124489796 | 2.070068027±0.097817358 |

Fig. 2

Fig.2A dsG6PD（Knockdown efficiency of G6PD)

| **G6PD** | **dsEGFP+WSSV** | | | **Mean±SD** | **dsG6PD+WSSV** | | | **Mean±SD** |
| --- | --- | --- | --- | --- | --- | --- | --- | --- |
| 24h | 1.225468443 | 0.872564288 | 0.935191248 | 1.01107466±0.188292471 | 0.323835563 | 0.364333948 | 0.254076233 | 0.314081915±0.055772226 |
| 48h | 1.30435207 | 1.002313162 | 0.764894847 | 1.02385336±0.270372907 | 0.165320018 | 0.099672086 | 0.037768658 | 0.100920254±0.06378484 |

| **TKTL2** | **dsEGFP+WSSV** | | | **Mean±SD** | **dsTKTL2+WSSV** | | | **Mean±SD** |
| --- | --- | --- | --- | --- | --- | --- | --- | --- |
| 24h | 1.180992661 | 0.901250463 | 0.939522749 | 1.007255291±0.151672995 | 0.162667732 | 0.156041319 | 0.181746565 | 0.166818539±0.013345854 |
| 48h | 0.957050307 | 0.977159968 | 1.069299999 | 1.001170091±0.059852843 | 0.052799448 | 0.051712851 | 0.047916511 | 0.050809603±0.00256372 |

Fig.2B dsTKTL2（Knockdown efficiency of TKTL2)

Fig.2C dsG6PD（viral load）

| **viral load** | **dsEGFP+WSSV** | | | **Mean±SD** | **dsG6PD+WSSV** | | | **Mean±SD** |
| --- | --- | --- | --- | --- | --- | --- | --- | --- |
| 24h | 67543.83103 | 66126.68304 | 87115.03252 | 73595.1822±11729.95489 | 26570.01756 | 25109.3019 | 18659.90048 | 23446.40665±4209.08583 |
| 48h | 1305476.906 | 1451489.669 | 1391220.751 | 1382729.109±73375.83207 | 256891.4258 | 269921.303 | 124043.9597 | 216952.2295±80724.24898 |

| **viral load** | **dsEGFP+WSSV** | | | **Mean±SD** | **dsTKTL2+WSSV** | | | **Mean±SD** |
| --- | --- | --- | --- | --- | --- | --- | --- | --- |
| 24h | 5813.223393 | 4507.228745 | 4735.841424 | 5018.764521±697.4522307 | 36.34193516 | 43.67359664 | 31.10826151 | 37.04126444±6.311791173 |
| 48h | 40318.20726 | 39472.28446 | 39194.27285 | 39661.58819±585.3922327 | 327.3929425 | 262.9723891 | 259.2810944 | 283.2154753±38.30330101 |

Fig.2D dsTKTL2（viral load）

Fig.2E dsG6PD（The mRNA levels of the viral gene IE1）

| IE1 | **dsEGFP+WSSV** | | | **Mean±SD** | **dsG6PD+WSSV** | | | **Mean±SD** |
| --- | --- | --- | --- | --- | --- | --- | --- | --- |
| 24h | 1.104454001 | 0.981685855 | 0.922316194 | 1.002818683±0.092889679 | 0.364333948 | 0.326088017 | 0.321598667 | 0.337340211±0.023484781 |
| 48h | 1.203025036 | 0.983956654 | 0.844791174 | 1.010590955±0.180595998 | 0.313528102 | 0.37805464 | 0.288504688 | 0.32669581±0.046204327 |

| IE1 | **dsEGFP+WSSV** | | | **Mean±SD** | **dsTKTL2+WSSV** | | | **Mean±SD** |
| --- | --- | --- | --- | --- | --- | --- | --- | --- |
| 24h | 1.228303149 | 0.905424761 | 0.899170536 | 1.010966149±0.188245339 | 0.099212566 | 0.077840601 | 0.08518048 | 0.087411216±0.010859206 |
| 48h | 0.903335201 | 0.866537046 | 1.277508892 | 1.015793713±0.227397564 | 0.077660959 | 0.066216443 | 0.066216443 | 0.070031282±0.006607494 |

Fig.2F dsTKTL2（The mRNA levels of the viral gene IE1）

Fig.2G dsG6PD（The mRNA levels of the viral gene VP28）

| VP28 | **dsEGFP+WSSV** | | | **Mean±SD** | **dsG6PD+WSSV** | | | **WSSV** |
| --- | --- | --- | --- | --- | --- | --- | --- | --- |
| 24h | 1.112136086 | 0.854607174 | 1.052144848 | 1.006296036±0.134747432 | 0.106087996 | 0.079844306 | 0.104627452 | 0.096853251±0.01474827 |
| 48h | 1.175547906 | 1.191957944 | 0.713672127 | 1.027059326±0.271525274 | 0.356836064 | 0.347078363 | 0.461158097 | 0.388357508±0.063235651 |

Fig.2H dsTKTL2（The mRNA levels of the viral gene VP28）

| VP28 | **dsEGFP+WSSV** | | | **Mean±SD** | **dsTKTL2+WSSV** | | | **WSSV** |
| --- | --- | --- | --- | --- | --- | --- | --- | --- |
| 24h | 1.394743666 | 0.933032992 | 0.768437591 | 1.032071416±0.324686436 | 0.032352029 | 0.027204705 | 0.027204705 | 0.02892048±0.002971809 |
| 48h | 0.903335201 | 1.143402487 | 0.968170696 | 1.004969461±0.124192139 | 0.120185382 | 0.10247425 | 0.098299871 | 0.106986501±0.011619561 |

Fig.2K Survival rate

|  | dsEGFP+PBS | dsTKTL2+PBS | dsG6PD+PBS | dsEGFP+WSSV | dsTKTL2+WSSV | dsG6PD+WSSV |
| --- | --- | --- | --- | --- | --- | --- |
| 0h | 100 | 100 | 100 | 100 | 100 | 100 |
| 12h | 100 | 100 | 100 | 97.5 | 100 | 97.5 |
| 24h | 97.4358974358974 | 97.4358974358974 | 100 | 85 | 100 | 95 |
| 36h | 97.4358974358974 | 97.4358974358974 | 100 | 67.5 | 97.3684210526316 | 95 |
| 48h | 97.4358974358974 | 97.4358974358974 | 100 | 47.5 | 84.2105263157895 | 82.1621621621622 |
| 60h | 97.4358974358974 | 97.4358974358974 | 100 | 30 | 65.7894736842105 | 69.3243243243243 |
| 72h | 97.4358974358974 | 94.6520146520146 | 100 | 7.5 | 57.8947368421053 | 51.3513513513513 |

Fig.2L Inhibitor treatment （cck8）

| **CCK8** |  | | | **Mean±SD** |
| --- | --- | --- | --- | --- |
| DMSO | 105.08 | 94.92 | 100 | 100±5.08 |
| **OT** | 101.69 | 83.05 | 98.31 | 94.35±9.930941547 |
| 6An | 81.36 | 79.66 | 89.83 | 83.61666667±5.447626394 |

Fig.2M Inhibitor treatment（viral load）

| viral load |  | | | **Mean±SD** |
| --- | --- | --- | --- | --- |
| DMSO+WSSV | 1605.981898 | 1943.664066 | 2100.812824 | 1883.486263±252.8446902 |
| OT+WSSV | 692.5517837 | 925.3540225 | 972.289218 | 863.3983414±149.807002 |
| DMSO+WSSV | 1308.3405 | 1836.809017 | 1434.254562 | 1526.468026±276.0384676 |
| 6An+WSSV | 456.3971045 | 601.2557529 | 540.7723644 | 532.8084073±72.75696162 |

Fig. 3

| Disatance | IE1 | TKTL2 | Disatance | IE1 | TKTL2 | Disatance | IE1 | TKTL2 | Disatance | IE1 | TKTL2 | Disatance | IE1 | TKTL2 |
| --- | --- | --- | --- | --- | --- | --- | --- | --- | --- | --- | --- | --- | --- | --- |
| 0 | 96 | 94 | 4.3333 | 187.278 | 154.228 | 8.6667 | 167.112 | 180.979 | 13 | 155.368 | 142.119 | 17.3333 | 84.491 | 129.402 |
| 0.1111 | 120.341 | 113.161 | 4.4444 | 214.373 | 179.85 | 8.7778 | 154.497 | 175.918 | 13.1111 | 159.495 | 154.039 | 17.4444 | 90.149 | 146.327 |
| 0.2222 | 118.667 | 104.746 | 4.5556 | 244.301 | 204.301 | 8.8889 | 154.216 | 181.076 | 13.2222 | 149.785 | 153.102 | 17.5556 | 84.941 | 148.662 |
| 0.3333 | 107.381 | 99.007 | 4.6667 | 249.168 | 192.855 | 9 | 132.663 | 171.053 | 13.3333 | 142.732 | 148.459 | 17.6667 | 68.992 | 142.185 |
| 0.4444 | 102.287 | 110.092 | 4.7778 | 240.507 | 170.691 | 9.1111 | 152.074 | 193.596 | 13.4444 | 123.951 | 139.162 | 17.7778 | 98.549 | 157.511 |
| 0.5556 | 99.194 | 121.731 | 4.8889 | 236.651 | 165.747 | 9.2222 | 127.724 | 177.362 | 13.5556 | 110.004 | 131.623 | 17.8889 | 98.621 | 136.204 |
| 0.6667 | 137.698 | 166.208 | 5 | 246.147 | 185.497 | 9.3333 | 156.733 | 187.374 | 13.6667 | 136.288 | 177.811 | 18 | 113.402 | 142.2 |
| 0.7778 | 168.476 | 204.502 | 5.1111 | 196.445 | 132.418 | 9.4444 | 215.881 | 209.379 | 13.7778 | 134.244 | 190.667 | 18.1111 | 119.015 | 140.368 |
| 0.8889 | 172.479 | 210.451 | 5.2222 | 198.854 | 144.339 | 9.5556 | 216.09 | 198.522 | 13.8889 | 127.958 | 171.283 | 18.2222 | 130.202 | 145.828 |
| 1 | 159.204 | 195.102 | 5.3333 | 214.813 | 180.634 | 9.6667 | 208.742 | 192.984 | 14 | 118.392 | 141.142 | 18.3333 | 138.836 | 153.029 |
| 1.1111 | 170.008 | 206.846 | 5.4444 | 218.473 | 210.364 | 9.7778 | 227.314 | 198.38 | 14.1111 | 111.74 | 133.415 | 18.4444 | 102.42 | 125.034 |
| 1.2222 | 160.226 | 198.127 | 5.5556 | 204.43 | 211.299 | 9.8889 | 239.828 | 192.434 | 14.2222 | 105.727 | 121.35 | 18.5556 | 109.362 | 120.553 |
| 1.3333 | 168.82 | 204.362 | 5.6667 | 193.691 | 198.379 | 10 | 242.831 | 195.264 | 14.3333 | 126.628 | 128.855 | 18.6667 | 164.863 | 157.506 |
| 1.4444 | 189.929 | 213.28 | 5.7778 | 189.294 | 191.54 | 10.1111 | 241.882 | 193.109 | 14.4444 | 137.118 | 123.169 | 18.7778 | 159.711 | 151.176 |
| 1.5556 | 196.647 | 197.697 | 5.8889 | 207.558 | 209.204 | 10.2222 | 239.452 | 206.013 | 14.5556 | 176.147 | 150.756 | 18.8889 | 145.546 | 145.927 |
| 1.6667 | 200.819 | 192.883 | 6 | 204.682 | 212.637 | 10.3333 | 236.074 | 221.002 | 14.6667 | 188.488 | 172.576 | 19 | 123.686 | 122.43 |
| 1.7778 | 194.495 | 187.277 | 6.1111 | 191.934 | 204.296 | 10.4444 | 222.519 | 220.636 | 14.7778 | 174.403 | 185.111 | 19.1111 | 121.362 | 107.178 |
| 1.8889 | 177.11 | 172.089 | 6.2222 | 212.049 | 223.026 | 10.5556 | 228.791 | 227.862 | 14.8889 | 154.009 | 189.764 | 19.2222 | 140.072 | 120.343 |
| 2 | 180.99 | 182.955 | 6.3333 | 218.985 | 222.89 | 10.6667 | 252.82 | 252.3 | 15 | 121.221 | 162.506 | 19.3333 | 150.551 | 129.304 |
| 2.1111 | 187.074 | 197.149 | 6.4444 | 207.405 | 211.221 | 10.7778 | 247.429 | 243.496 | 15.1111 | 127.271 | 161.669 | 19.4444 | 201.395 | 171.321 |
| 2.2222 | 201.483 | 213.025 | 6.5556 | 227.119 | 220.158 | 10.8889 | 254.43 | 249.76 | 15.2222 | 159.168 | 183.136 | 19.5556 | 235.647 | 184.14 |
| 2.3333 | 226.426 | 236 | 6.6667 | 231.141 | 219.365 | 11 | 245.56 | 237.239 | 15.3333 | 164.615 | 174.852 | 19.6667 | 247.287 | 174.106 |
| 2.4444 | 216.697 | 222.336 | 6.7778 | 239.106 | 220.354 | 11.1111 | 219.729 | 209.061 | 15.4444 | 160.865 | 165.029 | 19.7778 | 251.201 | 160.844 |
| 2.5556 | 229.776 | 226.543 | 6.8889 | 242.429 | 224.799 | 11.2222 | 202.655 | 201.052 | 15.5556 | 157.091 | 166.299 | 19.8889 | 235.764 | 155.236 |
| 2.6667 | 244.707 | 229.144 | 7 | 251.364 | 236.963 | 11.3333 | 222.858 | 241.135 | 15.6667 | 139.921 | 148.388 | 20 | 184.719 | 137.541 |
| 2.7778 | 224.571 | 179.883 | 7.1111 | 222.696 | 207.504 | 11.4444 | 185.335 | 215.69 | 15.7778 | 164.036 | 167.683 | 20.1111 | 135.305 | 111.69 |
| 2.8889 | 205.372 | 164.962 | 7.2222 | 225.117 | 217.602 | 11.5556 | 192.446 | 227.161 | 15.8889 | 181.751 | 193.107 | 20.2222 | 116.08 | 103.731 |
| 3 | 185.306 | 152.76 | 7.3333 | 172.22 | 181.593 | 11.6667 | 190.358 | 220.546 | 16 | 158.056 | 185.377 | 20.3333 | 118.813 | 100.019 |
| 3.1111 | 201.59 | 163.192 | 7.4444 | 150.989 | 169.84 | 11.7778 | 199.752 | 221.667 | 16.1111 | 151.054 | 187.076 | 20.4444 | 139.637 | 115.786 |
| 3.2222 | 207.56 | 166.714 | 7.5556 | 160.544 | 171.821 | 11.8889 | 207.706 | 223.938 | 16.2222 | 138.574 | 167.723 | 20.5556 | 147 | 130 |
| 3.3333 | 199.675 | 168.171 | 7.6667 | 192.188 | 187.004 | 12 | 163.195 | 180.577 | 16.3333 | 162.498 | 189.858 |  |  |  |
| 3.4444 | 190.738 | 170.841 | 7.7778 | 186.277 | 172.925 | 12.1111 | 186.283 | 212.64 | 16.4444 | 147.8 | 185 |  |  |  |
| 3.5556 | 241.667 | 220.693 | 7.8889 | 211.787 | 197.327 | 12.2222 | 206.893 | 243.801 | 16.5556 | 120.421 | 166.269 |  |  |  |
| 3.6667 | 216.998 | 187.213 | 8 | 189.282 | 183.821 | 12.3333 | 215.8 | 254.2 | 16.6667 | 128.193 | 167.898 |  |  |  |
| 3.7778 | 233.633 | 203.663 | 8.1111 | 179.903 | 186.618 | 12.4444 | 190.69 | 239.55 | 16.7778 | 123.736 | 159.343 |  |  |  |
| 3.8889 | 226.026 | 200.235 | 8.2222 | 197.4 | 197.2 | 12.5556 | 183.541 | 237.441 | 16.8889 | 136.671 | 168.286 |  |  |  |
| 4 | 218.284 | 194.958 | 8.3333 | 216.064 | 189.027 | 12.6667 | 174.011 | 210.24 | 17 | 83.641 | 109.788 |  |  |  |
| 4.1111 | 236 | 208.4 | 8.4444 | 230.235 | 187.317 | 12.7778 | 158.877 | 173.544 | 17.1111 | 94.534 | 122.083 |  |  |  |
| 4.2222 | 195.801 | 160.922 | 8.5556 | 201.243 | 178.815 | 12.8889 | 167.769 | 159.164 | 17.2222 | 82.37 | 115.934 |  |  |  |

Fig.3H fluorescence intensity profile

Fig.3I Pearson’s correlation coefficient analyses

| n | P |
| --- | --- |
| 1 | 0.875 |
| 2 | 0.885 |
| 3 | 0.969 |
| 4 | 0.948 |
| 5 | 0.941 |
| 6 | 0.971 |
| 7 | 0.952 |
| 8 | 0.969 |
| 9 | 0.948 |
| 10 | 0.953 |
| 11 | 0.974 |
| 12 | 0.844 |
| 13 | 0.875 |
| 14 | 0.863 |
| 15 | 0.862 |
| 16 | 0.853 |
| 17 | 0.862 |
| 18 | 0.853 |
| 19 | 0.885 |
| 20 | 0.969 |

Fig. 5

Fig.5B overexpression（Enzymatic activity of TKT）

| TKT activity |  | | | **Mean±SD** |
| --- | --- | --- | --- | --- |
| EV | 14.04808953 | 16.49987874 | 12.59026891 | 14.37941239±1.975751347 |
| IE1 | 17.36131819 | 18.95166795 | 19.68057825 | 18.66452146±1.185993944 |
| TKTL2 | 44.92738065 | 38.69851077 | 40.28886053 | 41.30491732±3.236353433 |
| IE1+TKTL2 | 84.5003652 | 88.72538346 | 86.45037363 | 86.55870743±2.114591446 |

Fig.5C dsIE1（Knockdown efficiency of IE1)

| **IE1** | **dsEGFP+WSSV** | | | **Mean±SD** | **dsG6PD+WSSV** | | | **Mean±SD** |
| --- | --- | --- | --- | --- | --- | --- | --- | --- |
| 24h | 0.895025071 | 1.205807828 | 0.926588062 | 1.00914032±0.171048639 | 0.26425451 | 0.174342958 | 0.228457863 | 0.222351777±0.045265716 |
| 36h | 1.175547906 | 0.897095409 | 0.948246031 | 1.006963115±0.148221862 | 0.118530754 | 0.062644573 | 0.0838139 | 0.088329742±0.028215438 |

Fig.5E dsIE1（Enzymatic activity of TKT）

| **TKTL2 activity** | **dsEGFP+WSSV** | | | **Mean±SD** | **dsG6PD+WSSV** | | | **Mean±SD** |
| --- | --- | --- | --- | --- | --- | --- | --- | --- |
| 24h | 98.76955523 | 96.35752476 | 104.4426059 | 99.85656196±4.150701385 | 78.23075035 | 74.15447509 | 75.24523013 | 75.87681852±2.110256601 |
| 36h | 61.74468807 | 68.00669024 | 61.27335457 | 63.67491096±3.758825962 | 32.96442818 | 28.87869624 | 28.97155378 | 30.2715594±2.332554893 |

Fig.5F *In vitro* TKT assay（Enzymatic activity of TKT）

| TKTL2 activity |  | | | **Mean±SD** |
| --- | --- | --- | --- | --- |
| GST | 4.951877111 | 6.140327618 | 5.843214991 | 5.645139907±0.618489253 |
| IE1 | 12.6768054 | 7.675409522 | 9.854235451 | 10.06881679±2.507593278 |
| GST+TKTL2 | 46.74571993 | 44.12122506 | 41.29865511 | 44.05520003±2.724132571 |
| IE1+TKTL2 | 89.62897571 | 82.59731021 | 87.54918732 | 86.59182441±3.612269055 |

Fig. 6

Fig.6B Overexpression（NADPH levels）

| NADPH |  | | | **Mean±SD** |
| --- | --- | --- | --- | --- |
| EV | 0.888510674 | 1.036595786 | 0.832340459 | 0.919148973±0.105518191 |
| IE1 | 1.34808516 | 1.327659628 | 1.317446861 | 1.331063883±0.015600258 |
| TKTL2 | 1.817872413 | 1.833191563 | 1.853617095 | 1.83489369±0.017933028 |
| IE1+TKTL2 | 2.103829871 | 2.124255404 | 2.175319236 | 2.13446817±0.036822653 |

Fig.6C Overexpression（R5P levels）

| R5P |  | | | **Mean±SD** |
| --- | --- | --- | --- | --- |
| EV | 1.125151883 | 1.033616849 | 1.137302552 | 1.098690428±0.056681899 |
| IE1 | 1.422033212 | 1.441879303 | 1.530984204 | 1.464965573±0.058028541 |
| TKTL2 | 1.409477521 | 1.328878088 | 1.449979749 | 1.396111786±0.061647265 |
| IE1+TKTL2 | 1.819360065 | 1.769137303 | 1.748076144 | 1.778857837±0.036622615 |

Fig.6D Overexpression（ROS measurement by microplate reader）

| ROS  （microplate reader） |  | | | Mean±SD |
| --- | --- | --- | --- | --- |
| EV | 1264.358 | 1215.571 | 1272.277 | 1250.735333±30.70953263 |
| IE1 | 1034.587 | 1042.889 | 1048.385 | 1041.953667±6.946390238 |
| TKTL2 | 1071.376 | 1045.653 | 1037.941 | 1051.656667±17.50736463 |
| IE1+TKTL2 | 859.782 | 864.752 | 819.857 | 848.1303333±24.61120189 |

Fig.6F Overexpression（ROS measurement by flow cytometry）

| ROS（flow cytometry） |  | | | Mean±SD |
| --- | --- | --- | --- | --- |
| EV | 65.6 | 72.3 | 71.2 | 69.7±3.593048845 |
| IE1 | 48.8 | 50.1 | 51.7 | 50.2±1.452583905 |
| TKTL2 | 41.8 | 41.3 | 49.7 | 44.26666667±4.712041313 |
| IE1+TKTL2 | 35.1 | 34.4 | 30.9 | 33.46666667±2.250185178 |

Fig.6G dsIE1（Knockdown efficiency of IE1)

| IE1 | dsEGFP+WSSV | | | Mean±SD | dsIE1+WSSV | | | Mean±SD |
| --- | --- | --- | --- | --- | --- | --- | --- | --- |
| 24h | 1.140763716 | 0.901250463 | 0.972654947 | 1.004889709±0.122967311 | 0.23981603 | 0.217637641 | 0.258816231 | 0.238756634±0.020609726 |
| 36h | 1.194715135 | 1.047294123 | 0.79922115 | 1.013743469±0.199870227 | 0.06410882 | 0.056589048 | 0.069188049 | 0.063295306±0.006338774 |

Fig.6H dsIE1（NADPH levels）

| **NADPH** | dsEGFP+WSSV | | | Mean±SD | dsIE1+WSSV | | | Mean±SD |
| --- | --- | --- | --- | --- | --- | --- | --- | --- |
| 24h | 5.223829996 | 5.300425744 | 5.188085314 | 5.237447018±0.057394778 | 4.243404425 | 4.457872519 | 4.376170388 | 4.359149111±0.108242477 |
| 36h | 4.437446986 | 4.182127827 | 4.003404415 | 4.207659743±0.218144785 | 2.61446819 | 2.629787339 | 2.803404367 | 2.682553299±0.104940005 |

Fig.6I dsIE1（R5P levels）

| R5P | dsEGFP+WSSV | | | Mean±SD | dsIE1+WSSV | | | Mean±SD |
| --- | --- | --- | --- | --- | --- | --- | --- | --- |
| 24h | 1.023596939 | 1.103316327 | 0.93877551 | 1.021896259±0.082283591 | 0.718112245 | 0.705357143 | 0.788265306 | 0.737244898±0.044642857 |
| 36h | 1.415816327 | 1.518494898 | 1.479591837 | 1.471301021±0.051838938 | 0.283163265 | 0.335459184 | 0.278061224 | 0.298894558±0.031768485 |

Fig.6J dsIE1（ROS measurement by microplate reader）

| **ROS** | dsEGFP+WSSV | | | Mean±SD | dsIE1+WSSV | | | WSSV |
| --- | --- | --- | --- | --- | --- | --- | --- | --- |
| 24h | 176.65 | 183.546 | 188.345 | 182.847±5.878750462 | 217.41 | 232.779 | 236.353 | 228.8473333±10.06492992 |
| 36h | 67.135 | 70.452 | 78.148 | 71.91166667±5.649735599 | 145.507 | 167.318 | 124.681 | 145.8353333±21.3203962 |

Fig.6L dsIE1（ROS measurement by flow cytometry）

| **ROS** | dsEGFP+WSSV | | | Mean±SD | dsIE1+WSSV | | | WSSV |
| --- | --- | --- | --- | --- | --- | --- | --- | --- |
| 24h | 11.9 | 9.67 | 7.84 | 9.803333333±2.03328142 | 22.5 | 22.9 | 25.7 | 23.7±1.743559577 |
| 36h | 3.78 | 3.19 | 2.54 | 3.17±0.620241888 | 12.7 | 12.6 | 13.2 | 12.83333333±0.321455025 |

Fig.6N Inhibitor treatment（CCK8）

| CCK8 |  | | | Mean±SD |
| --- | --- | --- | --- | --- |
| DMSO | 105.03 | 107 | 87.96 | 99.99666667±10.47049346 |
| OT | 95.84 | 96.5 | 93.22 | 95.18666667±1.734858304 |
| 6An | 93.22 | 97.81 | 93.22 | 94.75±2.650037736 |

Fig.6O Inhibitor treatment（NADPH levels）

| NADPH |  | | | Mean±SD |
| --- | --- | --- | --- | --- |
| EV+DMSO | 2.221276685 | 2.420425629 | 2.2774469 | 2.306383071±0.102679365 |
| IE1+DMSO | 4.018723565 | 4.028936331 | 4.018723565 | 4.02212782±0.005896343 |
| IE1+OT | 2.890212882 | 2.95148948 | 2.72680862 | 2.856170327±0.116144496 |
| IE1+6An | 2.788085218 | 2.910638414 | 2.8238299 | 2.840851177±0.063024712 |

Fig.6P Inhibitor treatment（R5P levels）

| R5P |  | | | Mean±SD |
| --- | --- | --- | --- | --- |
| EV+DMSO | 1.304526749 | 1.345679012 | 1.201646091 | 1.283950617±0.074188297 |
| IE1+DMSO | 2.823045267 | 2.395061728 | 2.427983539 | 2.548696845±0.238162243 |
| IE1+OT | 1.24691358 | 1.251028807 | 1.316872428 | 1.271604938±0.039256757 |
| IE1+6An | 1.403292181 | 1.316872428 | 1.37037037 | 1.36351166±0.043616222 |

Fig.6Q Inhibitor treatment（ROS measurement by microplate reader）

| ROS  （microplate reader） |  | | | Mean±SD |
| --- | --- | --- | --- | --- |
| EV+DMSO | 1156.924 | 1316.609 | 1108.881 | 1194.138±108.7492154 |
| IE1+DMSO | 803.56 | 898.879 | 847.043 | 849.8273333±47.72046023 |
| IE1+OT | 1231.799 | 1155.651 | 1103.563 | 1163.671±64.49308664 |
| IE1+6An | 1138.057 | 1107.461 | 1203.081 | 1149.533±48.83205947 |

Fig.6S Inhibitor treatment（ROS measurement by flow cytometry）

| ROS（flow cytometry） |  | | | Mean±SD |
| --- | --- | --- | --- | --- |
| EV+DMSO | 82.2 | 84.5 | 80.7 | 82.46666667±1.913983629 |
| IE1+DMSO | 68.8 | 64.2 | 68.8 | 67.26666667±2.655811238 |
| IE1+OT | 83.5 | 84.2 | 86.5 | 84.73333333±1.569500982 |
| IE1+6An | 84.8 | 85.8 | 85.8 | 85.46666667±0.577350269 |

Fig. 7

Fig.7A R5P and NADPH supplementation（CCK8）

| CCK8 |  | | | **Mean±SD** |
| --- | --- | --- | --- | --- |
| H2O | 80.34 | 108.55 | 111.11 | 100±17.07410613 |
| NADPH | 113.68 | 108.55 | 105.98 | 109.4033333±3.920284854 |
| R5P | 116.24 | 95.73 | 100.85 | 104.2733333±10.67494418 |

Fig.7B R5P and NADPH supplementation（The mRNA levels of the viral gene IE1）

| IE1 |  | | | Mean±SD |
| --- | --- | --- | --- | --- |
| H2O+WSSV | 1.242575344 | 0.708742434 | 1.135504429 | 1.028940736±0.28242036 |
| NADPH+WSSV | 3.211691527 | 3.301984466 | 3.080860445 | 3.198178813±0.111179599 |
| R5P+WSSV | 3.059579387 | 2.757447338 | 2.663518559 | 2.826848428±0.206950304 |

Fig.7C R5P and NADPH supplementation（The mRNA levels of the viral gene VP28）

| VP28 |  | | | Mean±SD |
| --- | --- | --- | --- | --- |
| H2O+WSSV | 1.391524844 | 1.05457863 | 0.681443838 | 1.042515771±0.355194163 |
| NADPH+WSSV | 7.709656947 | 10.53170176 | 7.981537412 | 8.740965373±1.556769846 |
| R5P+WSSV | 8.378352983 | 5.229476989 | 6.665285471 | 6.757705148±1.576471076 |

Fig.7E R5P and NADPH supplementation（viral load）

| viral load |  | | | Mean±SD |
| --- | --- | --- | --- | --- |
| H2O+WSSV | 11138.45222 | 12039.01624 | 10232.67173 | 11136.7134±903.1735104 |
| NADPH+WSSV | 96858.52665 | 90888.93305 | 96858.52665 | 94868.66212±3446.546472 |
| R5P+WSSV | 43271.07819 | 49490.41251 | 37566.84383 | 43442.77818±5963.638418 |

Fig.7F dsIE1+ supplementation（The mRNA levels of the viral gene IE1）

| IE1 |  | | | Mean±SD |
| --- | --- | --- | --- | --- |
| dsEGFP+H2O | 0.92444966 | 1.362887677 | 0.793700526 | 1.027012621±0.298132309 |
| dsIE1+H2O | 0.161171289 | 0.173940606 | 0.282567347 | 0.205893081±0.066708104 |
| dsIE1+R5P | 1.05457863 | 1.122462048 | 1.576436072 | 1.251158917±0.283735723 |
| dsIE1+NADPH | 1.203025036 | 1.450617005 | 1.481097552 | 1.378246531±0.152509656 |
| dsIE1+NAC | 1.353473524 | 1.245449622 | 1.061913804 | 1.220278983±0.147400605 |

Fig.7G dsIE1+ supplementation（The mRNA levels of the viral gene VP28）

| VP28 |  | | | **Mean±SD** |
| --- | --- | --- | --- | --- |
| dsEGFP+H2O | 0.981685855 | 1.167427804 | 0.872564288 | 1.007225982±0.149081678 |
| dsIE1+H2O | 0.436282144 | 0.522438576 | 0.616996125 | 0.525238948±0.090389531 |
| dsIE1+R5P | 1.794190818 | 1.25992105 | 1.769489662 | 1.607867177±0.301583184 |
| dsIE1+NADPH | 1.023373892 | 1.508728627 | 1.25992105 | 1.264007856±0.242703175 |
| dsIE1+NAC | 1.447269237 | 1.127660927 | 1.295342252 | 1.290090805±0.159868856 |

Fig.7I dsIE1+ supplementation（viral load）

| viral load |  | | | Mean±SD |
| --- | --- | --- | --- | --- |
| dsEGFP+H2O | 3519.425987 | 3302.516402 | 3325.94171 | 3382.628033±119.0480845 |
| dsIE1+H2O | 52.85663589 | 72.64969329 | 58.76845509 | 61.42492809±10.16040929 |
| dsIE1+R5P | 5813.223393 | 4507.228745 | 4735.841424 | 5018.764521±697.4522307 |
| dsIE1+NADPH | 9807.789579 | 9602.010767 | 9534.381782 | 9648.060709±142.4022604 |
| dsIE1+NAC | 8636.095438 | 8103.834812 | 7085.446495 | 7941.792248±787.9221919 |

Fig. 8

Fig.8C Protein blocking（CCK8）

| CCK8 | 24h | | | Mean±SD |
| --- | --- | --- | --- | --- |
| GST | 93.42 | 100 | 106.58 | 100±6.58 |
| TKTL2-C1-GST | 119.75 | 85.89 | 120.69 | 108.7766667±19.82600649 |
| TKTL2-C2-GST | 117.87 | 88.71 | 93.42 | 100±15.65403143 |
| TKTL2-C3-GST | 84.95 | 119.75 | 86.83 | 97.17666667±19.5716666 |

Fig.8D Protein blocking（NADPH levels）

| NADPH | 24h | | | Mean±SD |
| --- | --- | --- | --- | --- |
| GST | 1.669787301 | 1.557446871 | 1.516595805 | 1.581276659±0.079327188 |
| TKTL2-C1-GST | 1.031489403 | 1.000851104 | 0.980425571 | 1.004255359±0.025701565 |
| TKTL2-C2-GST | 1.434893674 | 1.34808516 | 1.511489422 | 1.431489419±0.081755305 |
| TKTL2-C3-GST | 1.409361759 | 1.485957506 | 1.133617067 | 1.342978777±0.185313171 |

Fig.8E Protein blocking（R5P levels）

| R5P | 24h | | | Mean±SD |
| --- | --- | --- | --- | --- |
| GST | 1.359777704 | 1.032452138 | 1.179289775 | 1.190506539±0.163950811 |
| TKTL2-C1-GST | 0.253906748 | 0.256965865 | 0.2630841 | 0.257985571±0.004672879 |
| TKTL2-C2-GST | 1.006449639 | 0.926912586 | 0.9819767 | 0.971779642±0.040737215 |
| TKTL2-C3-GST | 1.298595355 | 1.26953374 | 1.274122416 | 1.280750504±0.015623477 |

Fig.8F Protein blocking（ROS measurement by microplate reader）

| ROS  （microplate reader） | 24h | | | Mean±SD |
| --- | --- | --- | --- | --- |
| GST | 447.571 | 464.938 | 563.67 | 492.0596667±62.62134662 |
| TKTL2-C1-GST | 1237.419 | 1167.796 | 748.644 | 1051.286333±264.3976683 |
| TKTL2-C2-GST | 603.69 | 567.376 | 529.854 | 566.9733333±36.91964693 |
| TKTL2-C3-GST | 502.674 | 558.27 | 616.639 | 559.1943333±56.98812245 |

Fig.8H Protein blocking（ROS measurement by flow cytometry）

| ROS  （flow cytometry） | 24h | | | Mean±SD |
| --- | --- | --- | --- | --- |
| EV+DMSO | 40.7 | 38.4 | 31.7 | 36.93333333±4.675824348 |
| IE1+DMSO | 59.3 | 61 | 60.7 | 60.33333333±0.907377173 |
| IE1+OT | 35.2 | 34.4 | 33.9 | 34.5±0.655743852 |
| IE1+6An | 32.6 | 31.5 | 31.2 | 31.76666667±0.73711148 |

Fig.8I Protein blocking（viral load）

| viral load | 24h | | | Mean±SD |
| --- | --- | --- | --- | --- |
| EV+DMSO | 294.4588135 | 284.2341826 | 250.2779559 | 276.3236507±23.1283253 |
| IE1+DMSO | 48.21632034 | 60.45369848 | 60.8825068 | 56.51750854±7.192236321 |
| IE1+OT | 486.3733089 | 313.7988958 | 325.0870451 | 375.0864166±96.54239937 |
| IE1+6An | 294.4588135 | 404.7238747 | 377.105026 | 358.7625714±57.37534454 |

Fig. S2

Fig.S2 Tissue distribution analysis（The mRNA levels of the TKTL2）

| TKTL2 |  | | | Mean±SD |
| --- | --- | --- | --- | --- |
| Nerve | 124.7878215 | 128.2960847 | 106.3984507 | 119.8274523±11.76140045 |
| Hemocytes | 51.38706444 | 52.10440113 | 71.67178266 | 58.38774941±11.50989998 |
| Heart | 38.94411244 | 47.61464045 | 37.87918363 | 41.47931217±5.339963309 |
| Intestine | 43.81440413 | 24.99094123 | 24.64688356 | 31.15074297±10.9684014 |
| Muscle | 10.43481656 | 8.018505295 | 8.897088687 | 9.116803514±1.223047827 |
| Stomach | 8.958972832 | 7.908112163 | 9.668776451 | 8.845287149±0.885820529 |
| Eyestalk | 9.021287416 | 8.417158786 | 7.327562322 | 8.255336175±0.858379923 |
| Gill | 2.450936885 | 3.792984125 | 3.490257151 | 3.244726054±0.70390826 |
| Hepatopancreas | 1.277508892 | 0.974904856 | 0.802922882 | 1.018445543±0.240270293 |
